# Prenatal Choline Supplement in a Maternal Obesity Model Modulates Offspring Hepatic Lipidomes

**DOI:** 10.3390/nu15040965

**Published:** 2023-02-15

**Authors:** Hunter W. Korsmo, Isma’il Kadam, Aziza Reaz, Rachel Bretter, Anjana Saxena, Caroline H. Johnson, Jorge Matias Caviglia, Xinyin Jiang

**Affiliations:** 1 PhD Program in Biochemistry, Graduate Center of the City University of New York, New York, NY 10016, USA; 2 Department of Health and Nutrition Sciences, Brooklyn College of the City University of New York, Brooklyn, NY 11210, USA; 3 Department of Biology, Brooklyn College of the City University of New York, New York, NY 11210, USA; 4 Yale School of Public Health, Yale University, New Haven, CT 06510, USA

**Keywords:** choline, maternal obesity, fetal programming, plasmalogen, MAFLD, insulinopathy

## Abstract

Maternal obesity during pregnancy adversely impacts offspring health, predisposing them to chronic metabolic diseases characterized by insulin resistance, dysregulated macronutrient metabolism, and lipid overload, such as metabolic-associated fatty liver disease (MAFLD). Choline is a semi-essential nutrient involved in lipid and one-carbon metabolism that is compromised during MAFLD progression. Here, we investigated under high-fat (HF) obesogenic feeding how maternal choline supplementation (CS) influenced the hepatic lipidome of mouse offspring. Our results demonstrate that maternal HF+CS increased relative abundance of a subclass of phospholipids called plasmalogens in the offspring liver at both embryonic day 17.5 and after 6 weeks of postnatal HF feeding. Consistent with the role of plasmalogens as sacrificial antioxidants, HF+CS embryos were presumably protected with lower oxidative stress. After postnatal HF feeding, the maternal HF+CS male offspring also had higher relative abundance of both sphingomyelin d42:2 and its side chain, nervonic acid (FA 24:1). Nervonic acid is exclusively metabolized in the peroxisome and is tied to plasmalogen synthesis. Altogether, this study demonstrates that under the influence of obesogenic diet, maternal CS modulates the fetal and postnatal hepatic lipidome of male offspring, favoring plasmalogen synthesis, an antioxidative response that may protect the mouse liver from damages due to HF feeding.

## 1. Introduction

The number of women who are obese entering pregnancies has been rising in recent decades [1]. Maternal obesity has been identified as the first threat and linked to the long-term programming of metabolic abnormalities in the offspring that may evolve into metabolic diseases, such as metabolic-associated fatty liver disease (MAFLD) [2,3,4,5].

MAFLD, a newly developed term that highlights the metabolic deregulation of non-alcoholic fatty liver disease, is the predominant insulinopathy (i.e., a disease related to chronic insulin dysregulation) associated with obesogenic diets [6,7]. It is characterized by liver steatosis, dysregulated macronutrient metabolism, oxidative stress, with or without inflammation and fibrosis [5,8,9,10]. The lipid profile in the spectrum of MAFLD pathogenesis changes as the disease progresses, including decreases in certain complex lipid species such as polyunsaturated fatty acids (PUFAs), sphingolipids, and many unique phospholipids as reported in previous studies [11,12,13,14]. However, it is largely unexplored whether maternal dietary intake during pregnancy modifies the offspring lipidome, thereby reducing the risk of MAFLD development later in life.

Choline, a semi-essential nutrient, is vital for fetal epigenetic programming and cognitive development in early life in both animals and humans [15,16,17,18,19,20]. Choline is essential for the synthesis of phosphatidylcholine (PC) and betaine and plays vital roles in both lipid and one-carbon metabolism in the liver [21,22]. PC is synthesized via the CDP-choline pathway, which uses an intact choline molecule as the starting material, and the de novo pathway which sequentially methylates phosphatidylethanolamine (PE) by the enzyme phosphatidylethanolamine N-methyltransferase (PEMT) [23]. The de novo pathway synthesizes more PC species with unsaturated fatty acids such as docosahexaenoic acid (DHA) than the CDP-choline pathway [23]. Moreover, choline promotes the transport of DHA, as DHA can be incorporated to a lyso-PC and then transported via the mammalian family super domain 2 a (MFSD2A) transporter [21,24,25]. The phosphocholine head group of PC is also integral for sphingomyelin (SM) synthesis. Taken together, choline is involved with multiple pathways of lipid metabolism and may thus influence the offspring lipidome.

We have previously demonstrated positive impacts of prenatal choline supplementation (CS) during maternal high-fat (HF) feeding. Prenatal CS not only reduces fetal liver triglyceride accumulation and overall adiposity but also improves glucose tolerance in male offspring during postnatal HF feeding in early adulthood [20,26,27,28]. In this study, we investigate how maternal HF+CS modulates the offspring lipidomic profile in both the fetal period and early adulthood. Of particular interest is the abundance of choline and ethanolamine-based phospholipids (PCs and PEs, respectively). 

## 2. Materials and Methods

### 2.1. Animals and Diets

The study protocol was approved by the Institutional Animal Care and Use Committee (IACUC) at Brooklyn College (approval code: #311, date: 3 May 2022). C57BL/6J mice were obtained from the Jackson Laboratory originally and were bred in the Brooklyn College animal facility. The mice were housed at 22 °C, humidity 40–60%, and a 12-h light/dark cycle with regular bedding and enrichment. The mice were fed a regular lab diet (Laboratory Rodent Diet 5012, LabDiet, St. Louis, MO, USA) ad libitum until experiments were initiated. F0 female mice were divided into three groups: the normal-fat control (NFCO) group received a normal-fat (NF) diet (D12450J, Research Diets, New Brunswick, NJ, USA) containing 10% kcal from fat and purified drinking water; the high-fat control (HFCO) group received a HF diet (D12492, Research Diets) containing 60% kcal from fat and purified drinking water; and the HF choline-supplemented (HFCS) group received the HF diet and purified drinking water supplemented with 25 mM of choline chloride. Male mice for mating received the NFCO diet. 

After 4 weeks of feeding with experimental diets, female and male mice (F0) were caged together in a 2:1 ratio for timed mating. If a vaginal plug was detected in the morning, the female mouse was transferred to a separate cage and time was recorded as embryonic day (E) 0.5. Female mice continued to receive their assigned diets during gestation. A subset of the mice was dissected at E17.5. The rest of the animals continued their dietary treatments until birth of pups. As HF dams were less successful in keeping their pups during lactation, to increase the survival rate of HF pups all female mice were provided with the NFCO diet during lactation until weaning of pups on postnatal day 21. After weaning, two male and two female offspring were randomly chosen from each litter and fed either a NF or HF diet without choline supplementation for 6 weeks. Each prenatal+postnatal dietary treatment group contained 6–8 F1 animals for each sex. This mouse model was well-established in previous studies of our lab and demonstrated significant impacts on offspring epigenetic modification and metabolic functions with prenatal HF feeding and CS [20,26,27,28]. The composition of experimental diets has been described previously [20,26,27,28]. The supplemental choline led to a 4.5-fold intake of choline in total compared to the non-supplemented control groups according to prior studies [20,26,27,28].

### 2.2. Sample Collection 

At E17.5, a subset of F0 females was euthanized by carbon dioxide inhalation after 6-h fasting. Thereafter, the fetuses were dissected, and the fetal livers were retrieved. For the other subset of animals, F1 offspring mice were euthanized by carbon dioxide inhalation after 6-h fasting following 6 weeks of post-weaning feeding. Livers were dissected, rinsed in phosphate buffered saline, and dried on absorbent paper. The samples were then weighed on an analytical balance. Thereafter, they were either flash-frozen in liquid nitrogen and stored at −80 °C or immersed in RNAlater^®^ (Thermo Scientific, Grand Island, NY, USA) overnight before being stored at −80 °C until analysis. 

### 2.3. Commercial Lipidomic Preparation and Detection

A subset of sex-pooled fetal liver samples (*n* = 5/maternal diet group) was randomly chosen from the groups and sent to the University of San Diego (UCSD) LIPID MAPS Lipidomics Core Facility for isobaric identification and relative concentration measurement of a phospholipid panel of 286 phospholipid species by LC-MS/MS. Samples were homogenized and extracted by the Bligh and Dyer method, dried down and reconstituted in 50 μL of buffer A (59:40:1 of isopropanol, hexane and water, respectively, with 10 mM ammonium acetate). Samples were injected at 10 μL and separated by normal phase ultra-high-performance liquid chromatography using a Waters ACQUITY UPLC System (Milford, MA, USA). Following separation, analytes were fragmented by a Sciex 6500 Qtrap mass spectrometer (Framingham, MA, USA) [29]. “Total” lipids are derived from the summation of individual concentrations of each measured lipid on the phospholipid panel. Resulting peak intensities associated with lipids are normalized by protein abundance measured with the Bradford assay. Total PE-to-PC ratios are derived from total lipids by dividing the relative abundance of PE by PC.

A subset of male offspring liver samples (*n* = 5/maternal diet group) after 6-week post-weaning HF feeding was randomly chosen and sent to the West Coast Metabolomics Center (WCMC) to measure a complex lipidomic panel. Samples were normalized by internal standards, and data were generated by both positive (Agilent 6530a QTOF) and negative (Agilent 6550 QTOF) modes by CSH-QTOF MS/MS [30]. The reason that only male offspring were included was because female offspring demonstrated no differences in blood glucose control, liver gene expression or lipid metabolism among the groups, while male offspring demonstrated improved glucose tolerance in the prenatally CS group at this time point based on our prior study [28]. Resulting peak intensities associated with lipids were normalized by tissue wet weight.

### 2.4. Lipidomic Data Processing

Both the prenatal and post-weaning lipidomics datasets were analyzed via MetaboAnalyst (http://www.metaboanalyst.ca, version 5.0, Xia Lab, McGill University, Montreal, Canada, accessed on 10 October 2022) after applying the log transformation and auto-scaling features and normalizing to the NFCO-NF group (prenatal and postnatal control diet without CS) for relative abundance comparison [31,32]. Sparse partial least squares discriminant analysis (sPLS-DA) was performed to determine lipid species that separated the treatment groups. Cross-validation (CV) of sPLS-DA performance was conducted using five components and five-fold CV. Between-group analysis was conducted with univariate analysis of variance (ANOVA) with an applied false discovery rate (FDR) (*p* < 0.05). For lipids identified as having different peak intensities among treatment groups, post-hoc pair-wise analyses using Fisher’s LSD test were further conducted. Heatmapping with data clustering was performed using Euclidean distance measuring and a Ward clustering algorithm. Pathway analysis was also conducted using the online resource Lipid Pathway Enrichment Analysis (LIPEA) (https://lipea.biotec.tu-dresden.de, accessed on 10 October 2022) with a Benjamini-corrected *p* value < 0.05 being considered as a differentially regulated pathway by treatments. 

### 2.5. RNA Extraction and Quantitative Real-Time PCR

RNA was extracted from one female and one male liver or gonadal fat sample from each litter using the TRIzol^®^ reagent (Thermo Scientific, Grand Island, NY, USA). Reverse transcription was conducted using the High-Capacity cDNA Reverse Transcription kit (Thermo Scientific) following the manufacturer’s instructions. Gene transcript abundance was analyzed by quantitative real-time PCR with SYBR green detection using the CFX384 Touch™ Real-Time PCR Detection System (Bio-Rad, Hercules, CA, USA) as previously described. Data were expressed as the fold difference of the gene of interest relative to the housekeeping gene, beta-actin (*Actb*), using the 2-ΔΔCt method [33]. All primers were designed using GeneRunner Version 3.01 (http://www.softpedia.com, accessed on 1 January 2022) (Supplementary Table S1). Expression of the following genes was analyzed: *Acer2* (alkaline ceramidase 2), *Agps* (peroxisomal alkyldihydroxyacetonephosphate synthase), *Ccl2* [MCP-1/chemokine (C-C motif) ligand 2], *Cers2* (ceramide synthase 2), *Col1a1* (COL1A1 collagen type I alpha 1 chain); *Cybb* (cytochrome b-245 beta), *Elovl1* (elongation of very long chain fatty acids 1), *Fads2* (fatty acid desaturase 2), *Far1* (fatty acyl-CoA reductase 1), *Gnpat* (glyceronephosphate O-acyltransferase), *Gpx4* (glutathione peroxidase 4), *Mfsd2a* (mammalian family super domain 2 a), *Sod* (superoxide dismutase), and *Tnfa* (tumor necrosis factor alpha). 

### 2.6. Hepatic Malondialdehyde Measurements

Lipid peroxide levels were measured from malondialdehyde (MDA) using the TBARs TCA Method Assay Kit (Cayman Chemical, Ann Arbor, MI, USA). Fetal and male offspring liver tissue samples (*n* = 6–8/maternal diet group) were weighed and then homogenized in 250 μL 1X RIPA Buffer with TritonTM X-100 (Fisher Scientific, Waltham, MA, USA) on ice. Homogenates were then centrifuged, supernatant collected, and assay performed as per the manufacturer’s protocol.

### 2.7. Serum Alanine Transaminase (ALT) Measurements

Serum ALT levels after 6-week postnatal HF feeding were measured with the ALT (SGPT) reagent set (Teco Diagnostics, Anaheim, CA, USA) following the manufacturer’s instructions. 

### 2.8. Statistical Analysis

Univariate ANOVA followed by post-hoc Fisher’s LSD tests were constructed in SPSS (version 24, IBM Inc., Armonk, NY, USA) to assess the differences among the dietary treatment groups. A *p* value < 0.05 was considered as significant. Values are presented as means ± standard error of mean (SEM). 

## 3. Results

### 3.1. Prenatal Choline Supplement during Maternal Obesity Modulates Fetal Hepatic Phospholipidomics and Lowers Oxidative Stress

**Figure 1:**
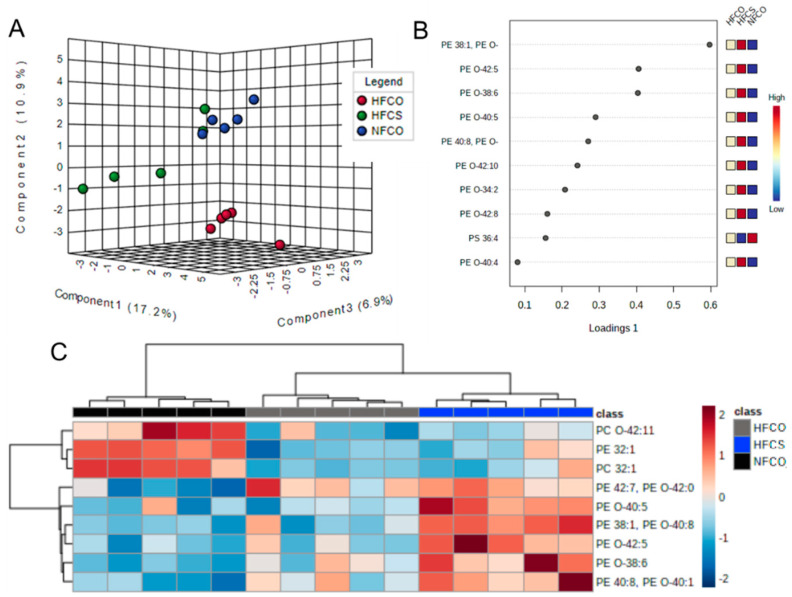
Phospholipid profile of Fetal (E17.5) livers in different maternal intake groups. (**A**) Sparse partial least squares discriminant analysis (sPLS-DA) scores plot. (**B**) Loadings plots of lipids that contributed to differences among groups. (**C**) Heatmap of phospholipids that differed among groups generated from Univariate ANOVA results with Euclidean distance measure and Ward clustering method (FDR-adjusted *p* < 0.05). CO, untreated control without choline supplement; CS, choline-supplemented; HF, high-fat; NF, normal-fat; PC, phosphatidylcholine; PE, phosphatidylethanolamine; O-, ether-linked fatty acid moiety.

We investigated the offspring hepatic lipidome at two time points, fetal E17.5 and male offspring after 6 weeks of HF feeding postweaning. At the fetal time point, 286 phospholipids including PE, phosphoinositol (PI), phosphatidylglycerol (PG), phosphatidylserine (PS), phosphatidic acid (PA), PC, and their lysophospholipids were detected. The normalized abundance of each lipid in different groups was presented in Supplementary Table S2. As demonstrated by sPLS-DA, there was separation among the three groups (Figure 1A), and PE species seem to have driven the differences (Figure 1B). Cross-validation of sPLS-DA results indicates a classification error rate of 20% (Supplementary Figure S1). ANOVA analyses identified nine phospholipids that had different concentrations among the groups (Figure 1C). Post-hoc pair-wise analyses found five lipid species that differed between the high-fat, choline-supplemented (HFCS) and high-fat without choline supplement (HFCO) groups after FDR adjustment for multiple testing. The five lipid species were all PEs, including PE 38:1 or PE O-40:8, PE 40:8 or PE O-40:1, PE O-38:6, PE O-42:5, and PE O-40:5, and all of them were significantly increased in the HFCS versus the HFCO fetal livers (Table 1). Phospholipids designated with “O-” indicate the presence of a fatty alcohol substituent forming an ether bond to the glycerol backbone, categorizing these lipids as plasmalogens. Plasmalogens play a consequential role as endogenous antioxidants by terminating products of free radicals (reactive oxygen species; ROS) intracellularly, in a polyunsaturated fatty acid (PUFA)-sparing manner [34,35].

**Figure 2:**
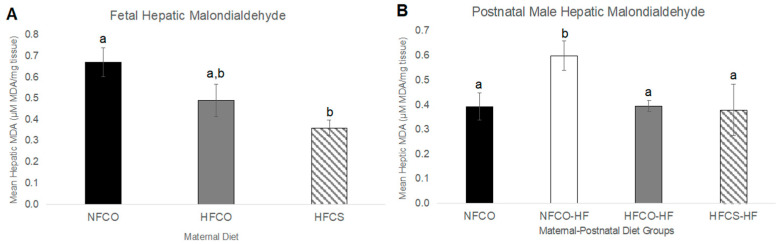
Hepatic lipoxidation in fetal and postnatal offspring livers. (**A**) E17.5 fetal liver lipoxidation measured with malondialdehyde (MDA) levels; (**B**) Male offspring liver lipoxidation after 6-week post-weaning HF feeding. Dams were fed NF or HF diets with or without CS prior to and during pregnancy. *n* = 6–10/group; values represent means ± SEM. a,b, different letters indicate significant differences between groups (*p* < 0.05) analyzed with ANOVA and Post-hoc Fisher’s LSD; any two groups with no overlapping letters demonstrated significant difference in their pair-wise comparison; CO, untreated control without choline supplement; CS, choline-supplemented; HF, high-fat; MDA, malondialdehyde; NF, normal-fat.

Plasmalogens may serve as sacrificial antioxidants to prevent other lipids from peroxidation. Therefore, we assessed PUFA lipoxidation by measuring malondialdehyde (MDA), a byproduct of PUFA lipoxidation, in the fetal livers [36,37,38]. Surprisingly, the prenatal high-fat control (HFCO) fetuses had numerically lower MDA levels than the prenatal normal-fat diet control (NFCO) fetuses, although they did not reach statistical significance (Figure 2A). When the fetuses were exposed to both maternal HF and choline supplement (HFCS), there was a significant decrease in hepatic MDA (*p* = 0.002) in prenatal HFCS fetal livers compared to the NFCO group (Figure 2A). Overall, it seems that the synergy of prenatal HF and CS was needed to significantly suppress PUFA lipoxidation in fetal (E17.5) livers. 

### 3.2. Prenatal Choline Supplement during Maternal Obesity Lowers Fetal Hepatic PE:PC Ratio

**Figure 3:**
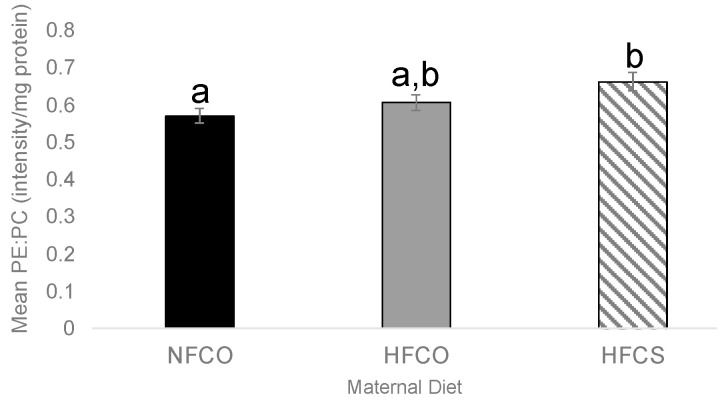
Fetal liver PE-to-PC ratios. Values represent means ± SEM. a,b, different letters indicate significant differences between groups (*p* < 0.05) analyzed with ANOVA and Post-hoc Fisher’s LSD; any two groups with no overlapping letters were significantly different in their pair-wise comparison; CO, untreated control without choline supplement; CS, choline-supplemented; HF, high-fat; NF, normal-fat; PC, phosphatidylcholine; PE, phosphatidylethanolamine.

We also examined the ratio of total PE:PC, which has been used as an indicator of cellular membrane integrity, in the E17.5 fetal liver. Interestingly, the HFCS group had a higher PE:PC ratio than the NFCO group (*p* = 0.031) (Figure 3), suggesting a synergy between HF and CS to increase this ratio.

### 3.3. Prenatal Choline Supplement during Maternal Obesity Modulates the Complex Lipidomic Profile of Male Offspring Livers

**Figure 4:**
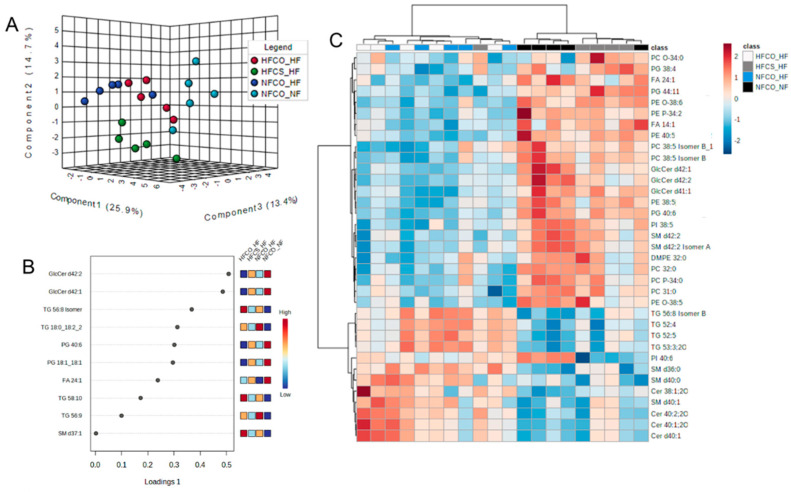
Lipidomics of male offspring livers after 6-week post-weaning HF feeding. (**A**) Sparse partial least squares discriminant analysis (sPLS-DA) scores plot of liver samples. (**B**) Loadings plots of lipids that contributed to differences among groups. (**C**) Heatmap of lipids that differed among groups generated from Univariate ANOVA results with Euclidean distance measure and Ward clustering method (FDR-adjusted *p* < 0.05). CO, untreated control without choline supplement; CS, choline-supplemented; HF, high-fat; NF, normal-fat; PC, phosphatidylcholine; PE, phosphatidylethanolamine; PG, phosphatidylglycerol; PI, phosphatidylinositol; Cer, ceramide; GlcCer, glucosylceramide; SM, sphingomyelin; DMPE, dimethylphosphatidylethanolamine; O-, ether linked fatty acid moiety.

**Figure 5:**
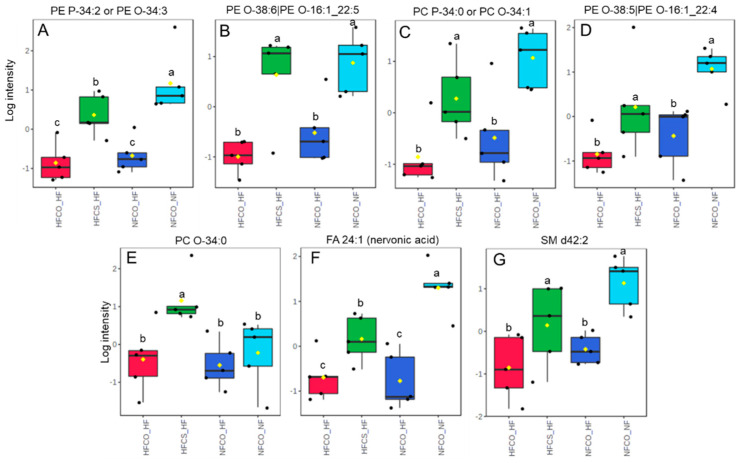
Phospholipids with differential abundance in the HFCS-HF versus other groups. (**A**) PE P34:2 or PE O-34:3; (**B**) PE O-38:6|PE O-16:1_22:5; (**C**) PC P-34:0 or PC O-34:1; (**D**) PE O-38:5|PE O-16:1_22:4; (**E**) PC O-34:0; (**F**) FA 24:1 (nervonic acid); (**G**) SM d42:2. Analyzed by MetaboAnalyst (FDR-adjusted *p* < 0.05). a,b,c any two groups with no overlapping letters were significantly different in their pair-wise comparison; HF, high-fat; CS, choline supplement; FDR: false discovery rate.

At the postnatal time point, the complex lipidome was compared among male offspring from different groups. There were 461 lipids including ceramides, sphingomyelins, cholesteryl esters, lyso- and phospholipids, mono-, di- and triacylglycerols that were identified from the lipidome analysis. The normalized abundance of each lipid in different groups was presented in Supplementary Table S3. As demonstrated by sPLS-DA, there was separation among NFCO-NF (the absolute control without HF or CS at any point in life), HFCS-HF (receiving HF both prenatally and postnatally but also CS during the prenatal period), and the other two groups: NFCO-HF which only received HF feeding postnatally and HFCO-HF, which received HF feeding both prenatally and postnatally (Figure 4A). The separation between NFCO-HF and HFCO-HF was not clear, suggesting that prenatal HF feeding itself did not substantially modify the lipidomic profile during postnatal life. The lipids with the highest loadings were not limited to a specific class of lipids (Figure 4B). However, cross-validation of the sPLS-DA suggests that the performance error rate was high at 55%, suggestive of potential model overfitting (Supplementary Figure S1). ANOVA analyses identified that 114 of the lipids were significantly different among treatment groups and 35 of them differed between HFCS-HF and HFCO-HF offspring in post-hoc pairwise tests (Figure 4C and Table 2). Similar to the prenatal time point, there was an increase in relative abundance of plasmalogens in HFCS-HF versus HFCO-HF samples (Figure 5). Some of them likely had identical composition as those identified in the fetal phospholipidomics. For example, PE O-38:6 identified in the fetal time point and PE (O-16:1/22:5) identified in the postnatal time point may be the same molecule. Interestingly, four out of five of these plasmalogens were downregulated in the postnatal HF groups (NFCO-HF and HFCO-HF) compared to the absolute normal-fat control (NFCO-NF), whereas maternal CS prevented their downregulation, affirming the persisting impact of maternal CS on offspring lipidome later in life against an adverse, obesogenic environment (Figure 5A–D). For PC O34:0, HFCS-HF had higher abundance compared to all other groups (Figure 5E). However, HFCS-HF postnatal male livers had no significant difference in oxidative stress levels as measured by MDA accumulation compared to other groups (Figure 2B). We also assessed glutathione abundance and confirmed its decrease by postnatal HF feeding. Maternal HFCS led to subtle and numerical alleviation of this decrease yet did not reach statistical significance (Supplementary Figure S2). 

There were also significant differences in the relative abundance of other complex lipid species: PEs, PCs and sphingolipids with LCFAs and VLCFAs (Figure 4C). VLCFA are fatty acids greater than 22 carbons in length. Nervonic acid (FA 24:1), a VLCFA, had greater relative abundance in the HFCS-HF versus HFCO-HF group (*p* = 0.027, Figure 5F). VLCFA breakdown occurs in the peroxisome and is required for the synthesis of plasmalogens [35]. Nervonic acid is mainly stored in hepatic sphingolipids, which are very abundant in the mouse liver [12,14,39,40]. Indeed, SM d42:2, which consists of the 18-carbon ceramide backbone being sphingosine and a VLCFA of 24 carbons, consistent with nervonic acid (FA 24:1), was elevated in relative abundance in HFCS-HF versus HFCO-HF offspring livers (*p* = 0.029, Figure 5G). In contrast, the other three SM species identified to have differential abundance, SM d36:0, SM d40:0, and SM d40:1 with LCFA substituents, were significantly lower in relative abundance in HFCS-HF compared to HFCO-HF (*p* = 0.038, *p* = 0.006, and *p* = 0.029, respectively, Post-hoc Fisher LSD). The data strongly suggest that prenatal CS impacts SM metabolism where VLCFA prevails predominantly.

Interestingly, many lipids in other lipid categories that were modulated by the diet, such as phosphatidylglycerols (PGs), phosphatidylinositols (PIs) and triglycerides (TGs), contain unsaturated substituents (Figure 4). Three PG species that contain PUFA side chains, PG (18:0/20:4), PG (18:1/22:5), and PG (22:5/22:6), were elevated in relative abundance in male HFCS-HF livers compared to HFCO-HF (Table 2) at the postnatal time point. PG metabolism is mediated predominantly in the mitochondria, possibly indicating mitochondrial changes [41]. Additionally, PI (18:0/22:6) had lower relative abundance, where PI 38:5 had a greater relative abundance in HFCS-HF compared to HFCO-HF (Table 2). Finally, four TG species, TG 52:4, TG 52:5, TG (16:0/18:2/19:1) and TG 56:8, were found to be significantly lower in relative abundance in HFCS-HF compared to the HFCO-HF group (Table 2).

We conducted pathway analysis on the 35 lipids with differential abundance in the HFCS-HF versus the HFCO-HF group. Results suggest that the pathways of sphingolipid metabolism, sphingolipid signaling, glycosylphosphatidylinositol (GPI)-anchor biosynthesis, autophagy, and necroptosis were significantly altered after Benjamini correction (Table 3). 

### 3.4. Prenatal Choline Supplement during Maternal Obesity Modulates Fetal and Male Offspring Hepatic mRNA Expression Related to Plasmalogen Synthesis and Lipid Metabolism

**Figure 6:**
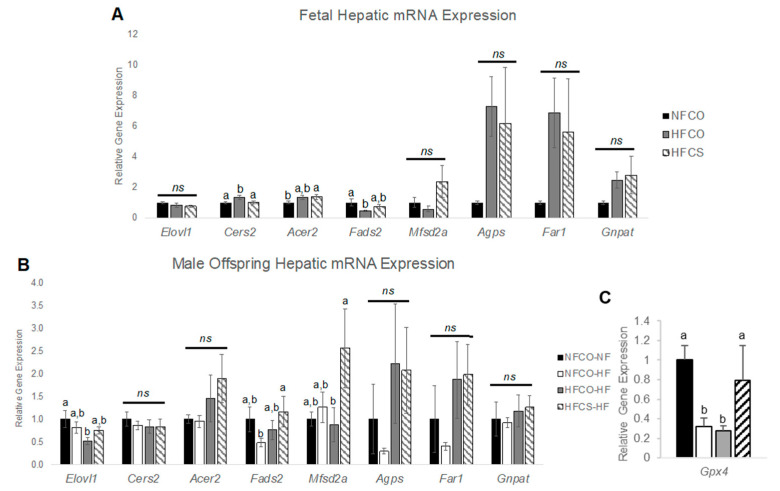
Fetal and Postnatal Hepatic Gene Expression of Proteins Involved in Lipid Metabolism. (**A**) Gene expression at E17.5; (**B**,**C**) Gene expression after 6-week postweaning HF feeding. *n* = 6–10/group; Values represent means ± SEM. a,b, different letters indicate significant differences between groups (*p* < 0.05) analyzed with ANOVA and Post-hoc Fisher’s LSD; any two groups with no overlapping letters were significantly different in their pair-wise comparison; ns, not significant; CO, untreated control without choline supplement; CS, choline-supplemented; HF, high-fat; NF, normal-fat. *Acer2*, alkaline ceramidase 2; *Agps*, alkylglycerone phosphate synthase; *Cers2*, ceramide synthase 2; *Elovl1*, elongation of very long chain fatty acid; *Fads2*, fatty acid desaturase 2; *Far1*, fatty acyl-CoA reductase 1; *Gnpat*, glyceronephosphate O-acyltransferase; *Gpx4*, glutathione peroxidase 4; *Mfsd2a*, mammalian family super domain 2a.

The lipidomic data suggest increases in both plasmalogens and lipids related to their synthesis, in the HFCS versus HFCO offspring. We therefore next examined mRNA expression of genes specific to plasmalogen metabolism in fetal and young adult male offspring livers. The de novo synthesis of plasmalogens in the peroxisome is catalyzed by the enzymes glyceronephosphate O-acyltransferase (*Gnpat*), fatty acyl-CoA reductase 1 (*Far1*), and alkylglycerone phosphate synthase (*Agps*). There were no significant differences found in hepatic gene expression (Figure 6).

Nervonic acid, which was also elevated in the HFCS-HF male offspring, is primarily synthesized by the enzyme elongation of very long chain fatty acid (*Elovl1*). Thereafter, nervonic acid can be incorporated into a hepatic pool of ceramides by ceramide synthase 2 (*Cers2*) and hydrolyzed from the sphingosine backbone of ceramide by alkaline ceramidase 2 (*Acer2*) where it is then catabolized in the peroxisome as an alkyl source for plasmalogen synthesis [13,39,42,43]. Our results demonstrate that maternal HFCS decreased the relative gene expression of *Cers2* compared to the HFCO group in the E17.5 fetal liver (*p* = 0.041; Post-hoc Fisher LSD; Figure 6A), which could be a feedback response to the higher abundance of PE plasmalogens in the HFCS group. There were no significant differences in fetal hepatic *Elovl1* or *Acer2* expression among the HFCS and HFCO groups.

Interestingly, during the postnatal time point, postnatal male hepatic relative gene expression of *Elovl1* was significantly decreased in the HFCO-HF group versus the negative control, NFCO-NF (*p* = 0.008; Post-hoc Fisher LSD; Figure 6B), consistent with the lower nervonic acid abundance in the HFCO-HF group (Figure 5F), indicating the role of prenatal plus postnatal HF feeding in decreasing the synthesis of VLCFAs. There was no difference in *Elovl1* expression between HFCS-HF and NFCO-NF offspring, suggesting an alleviation of *Elovl1* expression suppression by HF feeding with prenatal CS. Similarly, mRNA expression of other genes mediating nervonic acid metabolism did not differ among dietary treatment groups in the postnatal period. 

Because plasmalogens can incorporate PUFAs as substituents, we also measured expression of hepatic genes involved with PUFA synthesis and transport, i.e., *Fads2* and *Mfsd2a,* respectively, at both time points. We found that fetal hepatic *Fads2* was significantly decreased by the maternal HFCO diet compared to NFCO (*p* = 0.028; Post-hoc Fisher LSD; Figure 6A), while expression in the HFCS group was not significantly different from NFCO or HFCO. This data indicates that during maternal HF feeding the PUFA synthesis negatively impacted was probably alleviated with prenatal CS. Fetal hepatic *Mfsd2a* expression was not significantly different among groups. 

Postnatally, there were no significant differences in hepatic *Fads2* mRNA expression between HFCO-HF and HFCS-HF male offspring, yet there was a significant increase in relative expression of hepatic *Mfsd2a* in the HFCS-HF versus the HFCO-HF groups (*p* = 0.029; Post-hoc Fisher LSD; Figure 6B). *Mfsd2a* is critical for DHA transport. Prior studies have determined its upregulation in injured hepatocytes as a mechanism to promote hepatic regeneration following damage [44,45]. Thus, hepatic damage caused by continuous prenatal and postnatal HF feeding may be reversed via increases in *Mfsd2a* expression with prenatal CS.

Since plasmalogens may reduce oxidative stress and thereby reduce inflammation, we also measured genes in these pathways, such as *Gpx4*, *Sod*, and *Nrros,* which are negative regulators, and *Cybb*, which is a positive regulator of oxidative stress. We also measured *Tnfa* and *Ccl2*, which modulate inflammation and liver injury. There was no differential expression of the above genes for the E17.5 fetal liver and most of the 6-week postweaning HF-fed liver samples (Supplementary Figure S3). Only *Gpx4* was downregulated after 6 weeks of post-weaning HF feeding in both NFCO-HF and HFCO-HF groups compared to normal-fat control NFCO-NF, while this decrease was prevented for the HFCS-HF group (Figure 6C). There was also no difference in serum ALT level, an indicator of liver injury, among the groups at week 6 (Supplementary Figure S4).

## 4. Discussion

HF feeding during pregnancy poses obvious health risks for the fetuses as well as young offspring, including vulnerability to MAFLD development when offspring are challenged with an obesogenic environment after birth. In this study, with careful intervention during prenatal stages, we dissect how this can be reversed by maternal CS. Considering choline’s role in lipid biosynthesis and metabolism, we assessed both fetal and postnatal lipidomic profiles. We observed significant differences in the complex lipidomic profile between maternal HFCS-HF and HFCO-HF offspring, highlighting the increase in plasmalogens and lipids that regulate plasmalogen synthesis in the offspring from choline supplementeddams versus the maternal and postnatal HF-fed control (HFCO-HF). Molecularly speaking, plasmalogens contain a fatty alcohol, which forms an ether linkage, an important site for terminating PUFA peroxidation by radical propagation [35,46]. PUFAs are notoriously known for their susceptibility for bis-allylic attacks from radicals and increase the levels of MDA [38,47]. Plasmalogens as vinyl ether lipids serve to quench a lipophilic radical [48,49], thus sparing the PUFA from peroxidation [50,51,52]. MAFLD pathogenesis demonstrates chronic exhaustion of antioxidant potential due to the pro-oxidant strain induced from lipotoxic, hypercaloric (obesogenic) diets. Studies have demonstrated that an antioxidant treatment, e.g., tocopherol (vitamin E) treatment alleviates some key features of nonalcoholic steatohepatitis [53]. Plasmalogen synthesis decreases during MAFLD progression [11]. Therefore, the increase in plasmalogen levels in the HFCS-HF group may have positive implications in oxidative stress reduction during MAFLD pathogenesis. 

Although increased abundance of plasmalogens was observed in HFCS versus both HFCO (high-fat control) and NFCO (normal-fat control) fetal liver, decreases in MDA, indicative of lower lipid peroxidation, were only observed in the HFCS versus NFCO. It appears that both prenatal HF and CS were needed to mitigate lipid peroxidation. The potentially positive impact of prenatal HF on peroxidation reduction when combined with CS was unexpected, as other studies have demonstrated that HF feeding dysregulates redox balance [54,55]. At the postnatal time point, we did not observe significant differences in hepatic MDA despite greater plasmalogen abundance and the restoration of gene expression of *Gpx4*, a detoxifying enzyme involved in anti-oxidant response, in HFCS offspring versus HFCO. It is possible that there is a time-based effect, dependent on the length of the postnatal diet to induce significant lipid peroxidation that necessitates the increased use of plasmalogens. We previously reported that none of the groups demonstrated liver steatosis [20] and we also did not observe ALT elevation after 6-week postnatal HF feeding in this study, indicating that a longer exposure to HF feeding may be needed in future studies to fully examine the role of prenatal CS in plasmalogen metabolism and oxidative stress reduction during hepatic pathogenesis. Further research may be conducted after longer HF exposure to measure additional oxidative stress markers and an oxylipinomic panel to determine if there are specific reactive lipid species to demonstrate that more ether lipids, such as reactive aldehydes, are being peroxidized. The functional implications of plasmalogen increase in HFCS offspring are not clear. Literature has long established that pathology arising from obesity and lipotoxic diets is a consequence of chronically impaired insulin signaling [56,57,58]. Our previous findings of the positive influence of prenatal choline on male whole-body glucose tolerance may partly rely on this increased (antioxidant) plasmalogen species. Indeed, the integrity of the insulin receptor—and therefore the entire insulin response—relies on stable membranes and has been shown to be sensitive to oxidative stress (ROS) [57].

One of the metabolic substrates that promote plasmalogen synthesis is VLCFA, such as nervonic acid, which is exclusively catabolized in the peroxisome, where plasmalogen de novo synthesis initiates [35]. Breakdown of nervonic acid provides a source for the alkyl group in plasmalogen synthesis. Nervonic acid levels were reduced in the metabolic syndrome and its level in plasma was correlated with the level of plasmalogens [12]. A major source of murine hepatic VLCFAs is stored in sphingolipids [39]. Sphingomyelins, a downstream metabolite of choline via phosphocholine transfer from PC to a ceramide, are a major reservoir of FA 24 species and decrease along with plasmalogens and other complex lipids as MAFLD severity increases [59]. Briefly, elongation of very long chain fatty acid 1 (*Elovl1*) synthesizes FA 24 species, such as nervonic acid, which are then incorporated into ceramides by ceramide synthase 2 (*Cers2*), then metabolized to the choline derivative, SM. We observed a significant decrease in fetal hepatic *Cers2* expression by maternal HFCS which may be a feedback response to higher plasmalogen content in the HFCS fetus [13,60]. Further, the decrease in fetal hepatic *Cers2* in the HFCS group could be a result of increased ceramide catabolism as the reaction product sphingosine negatively regulates the expression of *Cers2*. The accumulation of ceramide, among other types of sphingolipids, is a trigger of lipotoxicity and insulin resistance [61].

**Figure 7:**
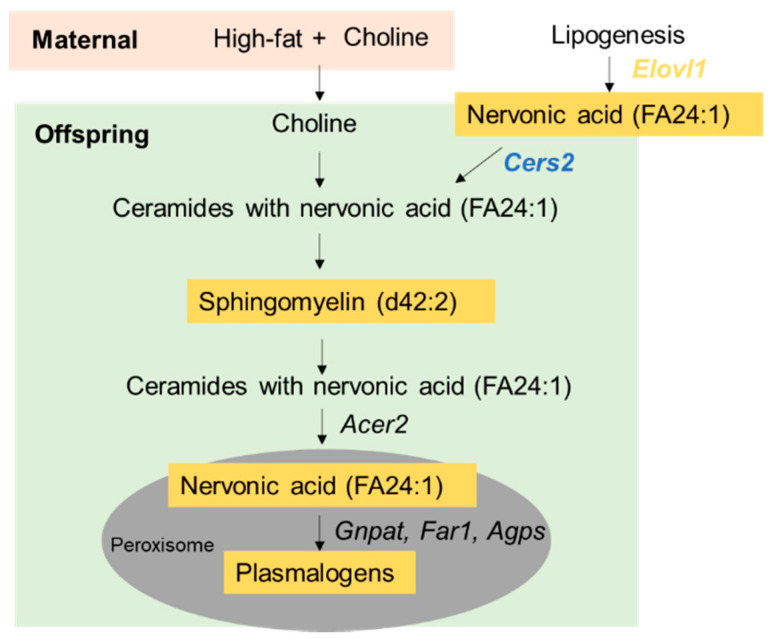
Potential mechanism by which maternal high-fat feeding and choline supplementation increase offspring hepatic plasmalogen abundance. Yellow highlight: increased abundance; blue highlight: decreased abundance by maternal high-fat plus choline supplementation in the current study. *Acer2*, alkaline ceramidase 2; *Agps*, alkylglycerone phosphate synthase; *Cers2*, ceramide synthase 2; *Elovl1*, elongation of very long chain fatty acid; FA, fatty acid; *Far1*, fatty acyl-CoA reductase 1; *Gnpat*, glyceronephosphate O-acyltransferase.

Sphingomyelinases (SMase) and subsequent alkaline ceramidase 2 (*Acer2*) actions can free up nervonic acid and other VLCFA from SM. SMase cleaves an SM to a ceramide and phosphocholine [62]. Thereafter, ACER2 releases VLCFA by hydrolyzing the ceramide backbone [43]. Indeed, we observed higher SM d42:2 relative abundance in the male HFCS offspring. Overall, the evidence suggests a potential mechanism of action by which maternal HFCS increases plasmalogen levels (Figure 7): HFCS may increase the substrate choline for SM synthesis. SM d42:2 (or 24:1, 18:1) may be cleaved into a nervonic acid-containing ceramide and further processed into nervonic acid. The available nervonic acid could then serve as a source of raw materials for plasmalogen synthesis. It should be noted that how maternal CS influences offspring plasmalogen synthesis may be different during the fetal versus postnatal periods. There was no difference in *Elovl1* expression in the fetal liver on E17.5 which indicated that maternal transport might be a major source of additional nervonic acid for the fetus. It is a limitation of this study that we do not have a measurement of maternal *Elovl1* expression or nervonic acid content to confirm the influence of HFCS on maternal metabolism. During postnatal HF feeding, there was no longer a direct impact of the maternal diet or nutrition supply on the offspring. *Elovl1* expression was downregulated in the HFCO-HF versus control offspring but partially restored in the HFCS-HF offspring livers, suggesting the activation of nervonic acid synthesis as a mechanism to preserve plasmalogen levels in the HFCS-HF group.

HFCS offspring further exhibited higher PUFA abundance in the PG, PI, and TG lipid classes. PGs, for instance, are generated in the mitochondria and play a critical role in MAFLD pathogenesis as PGs are involved in maintaining membrane potential and mitophagy [41]. There may be attributable differences in mitochondrial function due to prenatal HFCS, as indicated by differences in PGs. Since offspring mitochondria originate from mother’s ova in mammals, lipotoxic diets could influence the long-term mitochondrial function [63]. Omega-3 PUFAs are known to be depleted in the progression of MAFLD [64]. Previous studies strongly suggest that administering omega-3 PUFAs alleviates MAFLD pathology in pediatric MAFLD [65]. Although why maternal CS would increase PUFA abundance in other lipid classes is unclear, CS was reported to increase the incorporation of DHA into choline-containing phospholipids in pregnancy [66]. *Mfsd2a* encodes a membrane transporter of DHA and a marker of injury response [44]. Significantly increased expression of *Mfsd2a* in postnatal HFCS-HF versus HFCO-HF male offspring could possibly suggest increased activity in this pathway. Further studies are needed to determine whether choline status might be vital in improving DHA incorporation during diet-induced liver injury and inflammation. 

Choline supplementation in the HF dams increased the abundance of PE lipids and the PE: PC ratio in the fetal liver. These results were unexpected, since the literature suggests that choline supplementation increases the activity of PEMT, the enzyme that converts PE to PC, in both humans and rodents [67,68,69,70]. Moreover, the elevated PE:PC ratio is observed under the condition of choline deficiency and is associated with reduced cellular membrane fluidity and impaired cellular integrity [71]. A possible explanation for the higher PE:PC in HFCS fetal livers may be related to the homeostasis of several molecules in maintaining cellular membrane fluidity. In addition to PE and PC, membrane cholesterol also contributes to the regulation of membrane fluidity. Both PE and cholesterol reduce fluidity, while PC generally increases fluidity. If the level of cholesterol is diminished, the demand for PE is increased to prevent excessive fluidity of the membrane. A previous study demonstrated that increasing exogenous cholesterol content in the insect SF9 cells led to a decrease in the PE:PC ratio, while blocking sterol synthesis in the HEK 293T cells led to an increase in the PE:PC ratio [72]. Hence, it is possible that the HFCS fetal liver had lower cholesterol content, given that choline promotes lipid export from the liver. The PE level increased accordingly to maintain the viscosity of the cellular membrane. Further studies are needed to validate this hypothesis. It is also possible that choline supplementation increases PUFA-containing PC abundance on cellular membranes [69]. Since PUFAs increase membrane fluidity, the increase in PE serves as a compensatory mechanism to prevent excessive fluidity. Although the elevation in the PE:PC ratio is often viewed as a disturbance in membrane fluidity, a study suggests that this alteration could be beneficial for blood glucose control [73]. In *Pemt*^−/−^ female mice, PE levels are elevated. However, the increase in PE in these mice increased respiratory capacity, which increased the utilization of pyruvate for ATP production instead of using pyruvate as a substrate for hepatic glucose production via gluconeogenesis [73]. The study indicated that elevated PE may have a glucose-lowering effect by lowering gluconeogenesis. Thus, an increased PE:PC ratio under the HFCS model could possibly have a positive effect on glucose control, which requires further investigation. 

## 5. Conclusions

In conclusion, in this study we demonstrate that maternal CS during maternal HF feeding and diet-induced insulinopathy modify lipidomics in both fetal and postnatal livers. Specifically, maternal CS improves the relative abundance of anti-steatotic plasmalogen species, possibly by enhancing the release of nervonic acid from hepatic sphingolipid reservoirs for peroxisomal β-oxidation and plasmalogen synthesis. This proposed lipophilic antioxidant process may influence the offspring long-term insulin response by mitigating membrane damage in tissues.

## Figures and Tables

**Table 1:** Lipid species significantly differed between HFCS and the control groups.

Lipid Species	NFCO	HFCO	HFCS	FDR Adjusted *p* Value
PE 38:1, PE O-40:8	1 ^a^	1.29 ^a^	4.01 ^b^	0.005
PE 40:8, PE O-40:1	1 ^a^	1.94 ^b^	3.90 ^c^	0.033
PE O-38:6	1 ^a^	1.82 ^b^	4.05 ^c^	0.025
PE O-42:5	1 ^a^	1.30 ^a^	3.69 ^b^	0.033
PE O-40:5	1 ^a^	1.02 ^a^	3.28 ^b^	0.033

Data presented are fold changes compared to the normal diet control group NFCO. NF, normal-fat; HF, high-fat; CS, choline-supplemented; CO, untreated control without choline supplement; PE, phosphatidylethanolamine; FDR, false discovery rate; a, b, and c, different letters represent significant difference between groups in post-hoc pair-wise comparisons.

**Table 2:** Lipid species significantly differed between the maternal HFCS and HFCO offspring after 6-week post-weaning HF feeding.

Lipid Species	NFCO-NF	NFCO-HF	HFCO-HF	HFCS-HF	FDR Adjusted *p* Value
PG 40:6|PG 18:1_22:5	1 ^a^	0.26 ^c^	0.23 ^c^	0.59 ^b^	0.002
GlcCer d42:2	1 ^a^	0.25 ^c^	0.22 ^c^	0.41 ^b^	0.002
GlcCer d42:1	1 ^a^	0.24 ^c^	0.23 ^c^	0.44 ^b^	0.002
PE 38:5|PE 18:0_20:5	1 ^a^	0.28 ^b^	0.24 ^b^	0.65 ^a^	0.003
FA 24:1 (nervonic acid)	1 ^a^	0.24 ^c^	0.25 ^c^	0.45 ^b^	0.003
PE P-34:2 or PE O-34:3	1 ^a^	0.28 ^c^	0.25 ^c^	0.57 ^b^	0.004
GlcCer d41:1	1 ^a^	0.29 ^b^	0.28 ^b^	0.72 ^a^	0.006
SM d36:0	1^a^	4.17 ^c^	3.37 ^c^	1.83 ^b^	0.007
PC 32:0	1 ^a^	0.37 ^b^	0.22 ^b^	0.56 ^a^	0.007
TG 53:3;2O|TG 16:0_18:2_19:1;2O	1 ^a^	3.75 ^b^	3.68 ^b^	1.75 ^a^	0.008
TG 56:8 Isomer B	1 ^a^	3.77 ^c^	3.77 ^c^	1.93 ^b^	0.008
PI 38:5	1 ^a^	0.28 ^c^	0.26 ^c^	0.53 ^b^	0.008
SM d42:2 Isomer A	1 ^a^	0.31 ^b^	0.24 ^b^	0.48 ^a^	0.009
TG 52:5	1 ^a^	3.66 ^c^	3.80 ^c^	1.95 ^b^	0.009
PC 38:5 Isomer B_1	1 ^a^	0.41 ^b^	0.29 ^b^	0.71 ^a^	0.009
PE O-38:6|PE O-16:1_22:5	1 ^a^	0.38 ^b^	0.27 ^b^	0.85 ^a^	0.01
FA 14:1 (physeteric acid)	1 ^a^	0.27 ^b^	0.35 ^b^	0.74 ^a^	0.013
PE 40:5|PE 18:0_22:5	1 ^a^	0.28 ^b^	0.32 ^b^	0.67 ^a^	0.02
PG 38:4|PG 18:0_20:4	1 ^a^	0.41 ^b^	0.53 ^a,b^	1.51 ^c^	0.021
SM d42:2	1 ^a^	0.34 ^b^	0.25 ^b^	0.50 ^a^	0.021
TG 52:4	1 ^a^	3.44 ^c^	3.08 ^c^	1.47 ^b^	0.022
PC P-34:0 or PC O-34:1	1 ^a^	0.34 ^b^	0.26 ^b^	0.58 ^a^	0.022
PC 31:0	1 ^a^	0.33 ^b^	0.28 ^b^	0.61 ^a^	0.022
Cer d40:1	1 ^a^	2.37 ^b^	4.03 ^b^	1.73 ^a^	0.024
Cer 40:1;2O|Cer 18:1;2O/22:0	1 ^a^	2.20 ^b^	4.01 ^b^	1.76 ^a^	0.025
PE O-38:5|PE O-16:1_22:4	1 ^a^	0.35 ^b^	0.26 ^b^	0.55 ^a^	0.025
PI 40:6|PI 18:0_22:6	1 ^a^	0.50 ^b^	0.66 ^a^	0.25 ^a^	0.026
Cer 38:1;2O|Cer 18:1;2O/20:0	1 ^a^	2.25 ^b^	4.02 ^c^	1.91 ^a^	0.03
SM d40:0	1 ^a^	2.50 ^b^	2.70 ^b^	0.96 ^a^	0.033
Cer 40:2;2O|Cer 18:2;2O/22:0	1 ^a^	2.5 ^b^	3.70 ^b^	1.78 ^a^	0.04
PG 44:11|PG 22:5_22:6	1 ^a^	0.37 ^b^	0.41 ^b^	1.04 ^a^	0.043
DMPE 32:0|DMPE 16:0_16:0	1 ^a^	0.42 ^b^	0.30 ^b^	0.76 ^a^	0.044
PC 38:5 Isomer B_1	1 ^a^	0.41 ^b^	0.29 ^b^	0.71 ^a^	0.046
SM d40:1	1 ^a^	2.32 ^b^	3.64 ^b^	1.61 ^a^	0.046
PC O-34:0	1 ^a^	0.80 ^a^	0.89 ^a^	2.60 ^b^	0.049

Data presented are fold changes compared to the absolute control group that received normal fat during both prenatal and postnatal periods (NFCO-NF). a,b,c different letters indicate significant differences between groups (*p* < 0.05) analyzed with ANOVA and Post-hoc Fisher’s LSD; any two groups with no overlapping letters were significantly different in their pair-wise comparison; CO, untreated control without choline supplement; CS, choline-supplemented; FDR: false discovery rate; HF, high-fat; NF, normal-fat; PC, phosphatidylcholine; PE, phosphatidylethanolamine; PG, phosphatidylglycerol; PI, phosphatidylinositol; Cer, ceramide; GlcCer, glucosylceramide; SM, sphingomyelin; DMPE, dimethylphosphatidylethanolamine; O-, ether linked fatty acid moiety.

**Table 3:** Lipid pathways modified by maternal choline supplementation in male offspring mice after 6-week post-weaning high-fat feeding ^a^.

Pathway Name	Pathway Lipids (*n*)	Altered Lipids *(n*)	*p* Value after Benjamini Correction
Sphingolipid metabolism	21	5	0.004
Sphingolipid signaling pathway	9	3	0.014
Autophagy–other	3	2	0.014
Glycosylphosphatidylinositol (GPI)-anchor biosynthesis	3	2	0.014
Autophagy–animal	4	2	0.019
Necroptosis	4	2	0.019

^a^ Analyzed with LIPEA (Lipid Pathway Enrichment Analysis).

## Data Availability

Data will be available upon request.

## Supplementary Materials

The following supporting information can be downloaded at: https://www.mdpi.com/article/10.3390/nu15040965/s1. Table S1: Primers used for real-time PCR. Table S2: Abundance of lipid species in the E17.5 fetal liver. Table S3: Abundance of lipid species in male offspring livers after 6-week post-weaning high-fat feeding. Figure S1: Cross validation (CV) of Sparse partial least squares discriminant analysis (sPLS-DA) performance using 5-fold CV (A) at E17.5 and (B) after 6-week postweaning high-fat feeding of offspring. Figure S2: Abundance of glutathione in the liver of male offspring mice after 6-week postweaning HF feeding. Figure S3: Fetal and week 6 postweaning hepatic gene expression of proteins involved in oxidative stress regulation and inflammation. Figure S4: Serum alanine transaminase (ALT) concentrations after 6-week postweaning HF feeding.

## References

[1] Catalano P.M., Shankar K. (2017). Obesity and pregnancy: Mechanisms of short term and long term adverse consequences for mother and child. BMJ.

[2] Wesolowski S.R., Kasmi K.C., Jonscher K.R., Friedman J.E. (2017). Developmental origins of NAFLD: A womb with a clue. Nat. Rev. Gastroenterol. Hepatol..

[3] Nash M.J., Dobrinskikh E., Newsom S.A., Messaoudi I., Janssen R.C., Aagaard K.M., McCurdy C.E., Gannon M., Kievit P., Friedman J.E. (2021). Maternal Western diet exposure increases periportal fibrosis beginning in utero in nonhuman primate offspring. JCI Insight.

[4] Thompson M.D. (2020). Developmental Programming of NAFLD by Parental Obesity. Hepatol. Commun..

[5] Gutierrez Sanchez L.H., Tomita K., Guo Q., Furuta K., Alhuwaish H., Hirsova P., Baheti S., Alver B., Hlady R., Robertson K.D. (2018). Perinatal Nutritional Reprogramming of the Epigenome Promotes Subsequent Development of Nonalcoholic Steatohepatitis. Hepatol. Commun..

[6] Eslam M., Sanyal A.J., George J., Panel I.C. (2020). MAFLD: A Consensus-Driven Proposed Nomenclature for Metabolic Associated Fatty Liver Disease. Gastroenterology.

[7] Lambert J.E., Ramos-Roman M.A., Browning J.D., Parks E.J. (2014). Increased de novo lipogenesis is a distinct characteristic of individuals with nonalcoholic fatty liver disease. Gastroenterology.

[8] Larter C.Z., Yeh M.M., Cheng J., Williams J., Brown S., dela Pena A., Bell-Anderson K.S., Farrell G.C. (2008). Activation of peroxisome proliferator-activated receptor alpha by dietary fish oil attenuates steatosis, but does not prevent experimental steatohepatitis because of hepatic lipoperoxide accumulation. J. Gastroenterol. Hepatol..

[9] Wiering L., Tacke F. (2022). Treating inflammation to combat non-alcoholic fatty liver disease. J. Endocrinol..

[10] Zisser A., Ipsen D.H., Tveden-Nyborg P. (2021). Hepatic Stellate Cell Activation and Inactivation in NASH-Fibrosis-Roles as Putative Treatment Targets?. Biomedicines.

[11] Kartsoli S., Kostara C.E., Tsimihodimos V., Bairaktari E.T., Christodoulou D.K. (2020). Lipidomics in non-alcoholic fatty liver disease. World J. Hepatol..

[12] Yamazaki Y., Kondo K., Maeba R., Nishimukai M., Nezu T., Hara H. (2014). Proportion of nervonic acid in serum lipids is associated with serum plasmalogen levels and metabolic syndrome. J. Oleo Sci..

[13] Raichur S., Wang S.T., Chan P.W., Li Y., Ching J., Chaurasia B., Dogra S., Öhman M.K., Takeda K., Sugii S. (2014). CerS2 haploinsufficiency inhibits β-oxidation and confers susceptibility to diet-induced steatohepatitis and insulin resistance. Cell Metab..

[14] Konstantynowicz-Nowicka K., Berk K., Chabowski A., Kasacka I., Bielawiec P., Łukaszuk B., Harasim-Symbor E. (2019). High-Fat Feeding in Time-Dependent Manner Affects Metabolic Routes Leading to Nervonic Acid Synthesis in NAFLD. Int. J. Mol. Sci..

[15] Jiang X., Yan J., West A.A., Perry C.A., Malysheva O.V., Devapatla S., Pressman E., Vermeylen F., Caudill M.A. (2012). Maternal choline intake alters the epigenetic state of fetal cortisol-regulating genes in humans. FASEB J..

[16] Caudill M.A., Strupp B.J., Muscalu L., Nevins J.E.H., Canfield R.L. (2018). Maternal choline supplementation during the third trimester of pregnancy improves infant information processing speed: A randomized, double-blind, controlled feeding study. FASEB J..

[17] Meck W.H., Williams C.L. (2003). Metabolic imprinting of choline by its availability during gestation: Implications for memory and attentional processing across the lifespan. Neurosci. Biobehav. Rev..

[18] Blusztajn J.K., Mellott T.J. (2012). Choline nutrition programs brain development via DNA and histone methylation. Cent. Nerv. Syst. Agents Med. Chem..

[19] Kwan S.T.C., King J.H., Grenier J.K., Yan J., Jiang X., Roberson M.S., Caudill M.A. (2018). Maternal Choline Supplementation during Normal Murine Pregnancy Alters the Placental Epigenome: Results of an Exploratory Study. Nutrients.

[20] Korsmo H.W., Dave B., Trasino S., Saxena A., Liu J., Caviglia J.M., Edwards K., Dembitzer M., Sheeraz S., Khaldi S. (2022). Maternal Choline Supplementation and High-Fat Feeding Interact to Influence DNA Methylation in Offspring in a Time-Specific Manner. Front. Nutr..

[21] Korsmo H.W., Jiang X., Caudill M.A. (2019). Choline: Exploring the Growing Science on Its Benefits for Moms and Babies. Nutrients.

[22] Korsmo H.W., Jiang X. (2021). One carbon metabolism and early development: A diet-dependent destiny. Trends Endocrinol. Metab..

[23] DeLong C.J., Shen Y.J., Thomas M.J., Cui Z. (1999). Molecular distinction of phosphatidylcholine synthesis between the CDP-choline pathway and phosphatidylethanolamine methylation pathway. J. Biol. Chem..

[24] Wong B.H., Chan J.P., Cazenave-Gassiot A., Poh R.W., Foo J.C., Galam D.L., Ghosh S., Nguyen L.N., Barathi V.A., Yeo S.W. (2016). Mfsd2a Is a Transporter for the Essential ω-3 Fatty Acid Docosahexaenoic Acid (DHA) in Eye and Is Important for Photoreceptor Cell Development. J. Biol. Chem..

[25] Thomas Rajarethnem H., Megur Ramakrishna Bhat K., Jc M., Kumar Gopalkrishnan S., Mugundhu Gopalram R.B., Rai K.S. (2017). Combined Supplementation of Choline and Docosahexaenoic Acid during Pregnancy Enhances Neurodevelopment of Fetal Hippocampus. Neurol. Res. Int..

[26] Nam J., Greenwald E., Jack-Roberts C., Ajeeb T.T., Malysheva O.V., Caudill M.A., Axen K., Saxena A., Semernina E., Nanobashvili K. (2017). Choline prevents fetal overgrowth and normalizes placental fatty acid and glucose metabolism in a mouse model of maternal obesity. J. Nutr. Biochem..

[27] Jack-Roberts C., Joselit Y., Nanobashvili K., Bretter R., Malysheva O.V., Caudill M.A., Saxena A., Axen K., Gomaa A., Jiang X. (2017). Choline Supplementation Normalizes Fetal Adiposity and Reduces Lipogenic Gene Expression in a Mouse Model of Maternal Obesity. Nutrients.

[28] Korsmo H.W., Edwards K., Dave B., Jack-Roberts C., Yu H., Saxena A., Salvador M., Dembitzer M., Phagoora J., Jiang X. (2020). Prenatal Choline Supplementation during High-Fat Feeding Improves Long-Term Blood Glucose Control in Male Mouse Offspring. Nutrients.

[29] Quehenberger O., Armando A.M., Brown A.H., Milne S.B., Myers D.S., Merrill A.H., Bandyopadhyay S., Jones K.N., Kelly S., Shaner R.L. (2010). Lipidomics reveals a remarkable diversity of lipids in human plasma. J. Lipid. Res..

[30] Barupal D.K., Zhang Y., Shen T., Fan S., Roberts B.S., Fitzgerald P., Wancewicz B., Valdiviez L., Wohlgemuth G., Byram G. (2019). A Comprehensive Plasma Metabolomics Dataset for a Cohort of Mouse Knockouts within the International Mouse Phenotyping Consortium. Metabolites.

[31] Van den Berg R.A., Hoefsloot H.C., Westerhuis J.A., Smilde A.K., van der Werf M.J. (2006). Centering, scaling, and transformations: Improving the biological information content of metabolomics data. BMC Genom..

[32] Pang Z., Chong J., Zhou G., de Lima Morais D.A., Chang L., Barrette M., Gauthier C., Jacques P., Li S., Xia J. (2021). MetaboAnalyst 5.0: Narrowing the gap between raw spectra and functional insights. Nucleic Acids Res..

[33] Livak K.J., Schmittgen T.D. (2001). Analysis of relative gene expression data using real-time quantitative PCR and the 2(-Delta Delta C(T)) Method. Methods.

[34] Zoeller R.A., Lake A.C., Nagan N., Gaposchkin D.P., Legner M.A., Lieberthal W. (1999). Plasmalogens as endogenous antioxidants: Somatic cell mutants reveal the importance of the vinyl ether. Biochem. J..

[35] Engelmann B. (2004). Plasmalogens: Targets for oxidants and major lipophilic antioxidants. Biochem. Soc. Trans..

[36] Busch C.J., Hendrikx T., Weismann D., Jäckel S., Walenbergh S.M., Rendeiro A.F., Weißer J., Puhm F., Hladik A., Göderle L. (2017). Malondialdehyde epitopes are sterile mediators of hepatic inflammation in hypercholesterolemic mice. Hepatology.

[37] Busch C.J., Binder C.J. (2017). Malondialdehyde epitopes as mediators of sterile inflammation. Biochim. Biophys. Acta Mol. Cell Biol. Lipids.

[38] Hendrikx T., Binder C.J. (2020). Oxidation-Specific Epitopes in Non-Alcoholic Fatty Liver Disease. Front. Endocrinol..

[39] Ohno Y., Suto S., Yamanaka M., Mizutani Y., Mitsutake S., Igarashi Y., Sassa T., Kihara A. (2010). ELOVL1 production of C24 acyl-CoAs is linked to C24 sphingolipid synthesis. Proc. Natl. Acad. Sci. USA.

[40] Sztolsztener K., Konstantynowicz-Nowicka K., Harasim-Symbor E., Chabowski A. (2021). Time-Dependent Changes in Hepatic Sphingolipid Accumulation and PI3K/Akt/mTOR Signaling Pathway in a Rat Model of NAFLD. Int. J. Mol. Sci..

[41] Zhang X., Zhang J., Sun H., Liu X., Zheng Y., Xu D., Wang J., Jia D., Han X., Liu F. (2019). Defective Phosphatidylglycerol Remodeling Causes Hepatopathy, Linking Mitochondrial Dysfunction to Hepatosteatosis. Cell. Mol. Gastroenterol. Hepatol..

[42] Kihara A. (2012). Very long-chain fatty acids: Elongation, physiology and related disorders. J. Biochem..

[43] Sun W., Jin J., Xu R., Hu W., Szulc Z.M., Bielawski J., Obeid L.M., Mao C. (2010). Substrate specificity, membrane topology, and activity regulation of human alkaline ceramidase 2 (ACER2). J. Biol. Chem..

[44] Pu W., Zhang H., Huang X., Tian X., He L., Wang Y., Zhang L., Liu Q., Li Y., Zhao H. (2016). Mfsd2a+ hepatocytes repopulate the liver during injury and regeneration. Nat. Commun..

[45] Nguyen L.N., Ma D., Shui G., Wong P., Cazenave-Gassiot A., Zhang X., Wenk M.R., Goh E.L., Silver D.L. (2014). Mfsd2a is a transporter for the essential omega-3 fatty acid docosahexaenoic acid. Nature.

[46] Bozelli J.C., Azher S., Epand R.M. (2021). Plasmalogens and Chronic Inflammatory Diseases. Front. Physiol..

[47] Binder C.J., Papac-Milicevic N., Witztum J.L. (2016). Innate sensing of oxidation-specific epitopes in health and disease. Nat. Rev. Immunol..

[48] Stadelmann-Ingrand S., Favreliere S., Fauconneau B., Mauco G., Tallineau C. (2001). Plasmalogen degradation by oxidative stress: Production and disappearance of specific fatty aldehydes and fatty alpha-hydroxyaldehydes. Free Radic. Biol. Med..

[49] Stadelmann-Ingrand S., Pontcharraud R., Fauconneau B. (2004). Evidence for the reactivity of fatty aldehydes released from oxidized plasmalogens with phosphatidylethanolamine to form Schiff base adducts in rat brain homogenates. Chem. Phys. Lipids.

[50] Jang J.E., Park H.S., Yoo H.J., Baek I.J., Yoon J.E., Ko M.S., Kim A.R., Kim H.S., Lee S.E., Kim S.W. (2017). Protective role of endogenous plasmalogens against hepatic steatosis and steatohepatitis in mice. Hepatology.

[51] Almsherqi Z.A. (2021). Potential Role of Plasmalogens in the Modulation of Biomembrane Morphology. Front. Cell Dev. Biol..

[52] Sindelar P.J., Guan Z., Dallner G., Ernster L. (1999). The protective role of plasmalogens in iron-induced lipid peroxidation. Free Radic. Biol. Med..

[53] Sato K., Gosho M., Yamamoto T., Kobayashi Y., Ishii N., Ohashi T., Nakade Y., Ito K., Fukuzawa Y., Yoneda M. (2015). Vitamin E has a beneficial effect on nonalcoholic fatty liver disease: A meta-analysis of randomized controlled trials. Nutrition.

[54] Milagro F.I., Campión J., Martínez J.A. (2006). Weight gain induced by high-fat feeding involves increased liver oxidative stress. Obes..

[55] Tan B.L., Norhaizan M.E. (2019). Effect of High-Fat Diets on Oxidative Stress, Cellular Inflammatory Response and Cognitive Function. Nutrients.

[56] Rains J.L., Jain S.K. (2011). Oxidative stress, insulin signaling, and diabetes. Free Radic. Biol. Med..

[57] Hurrle S., Hsu W.H. (2017). The etiology of oxidative stress in insulin resistance. Biomed. J..

[58] Gill R., Tsung A., Billiar T. (2010). Linking oxidative stress to inflammation: Toll-like receptors. Free Radic. Biol. Med..

[59] Sassa T., Kihara A. (2014). Metabolism of very long-chain Fatty acids: Genes and pathophysiology. Biomol. Ther..

[60] Laviad E.L., Albee L., Pankova-Kholmyansky I., Epstein S., Park H., Merrill A.H., Futerman A.H. (2008). Characterization of ceramide synthase 2: Tissue distribution, substrate specificity, and inhibition by sphingosine 1-phosphate. J. Biol. Chem..

[61] Chaurasia B., Summers S.A. (2015). Ceramides-Lipotoxic Inducers of Metabolic Disorders. Trends Endocrinol. Metab..

[62] Insausti-Urkia N., Solsona-Vilarrasa E., Garcia-Ruiz C., Fernandez-Checa J.C. (2020). Sphingomyelinases and Liver Diseases. Biomolecules.

[63] Marei W.F.A., Smits A., Mohey-Elsaeed O., Pintelon I., Ginneberge D., Bols P.E.J., Moerloose K., Leroy J.L.M.R. (2020). Differential effects of high fat diet-induced obesity on oocyte mitochondrial functions in inbred and outbred mice. Sci. Rep..

[64] Monthé-Drèze C., Penfield-Cyr A., Smid M.C., Sen S. (2018). Maternal Pre-Pregnancy Obesity Attenuates Response to Omega-3 Fatty Acids Supplementation During Pregnancy. Nutrients.

[65] Chen L.H., Wang Y.F., Xu Q.H., Chen S.S. (2018). Omega-3 fatty acids as a treatment for non-alcoholic fatty liver disease in children: A systematic review and meta-analysis of randomized controlled trials. Clin. Nutr..

[66] Ferchaud-Roucher V., Kramer A., Silva E., Pantham P., Weintraub S.T., Jansson T., Powell T.L. (2019). A potential role for lysophosphatidylcholine in the delivery of long chain polyunsaturated fatty acids to the fetal circulation. Biochim. Biophys. Acta Mol. Cell Biol. Lipids.

[67] Yan J., Jiang X., West A.A., Perry C.A., Malysheva O.V., Brenna J.T., Stabler S.P., Allen R.H., Gregory J.F., Caudill M.A. (2013). Pregnancy alters choline dynamics: Results of a randomized trial using stable isotope methodology in pregnant and nonpregnant women. Am. J. Clin. Nutr..

[68] Yan J., Ginsberg S.D., Powers B., Alldred M.J., Saltzman A., Strupp B.J., Caudill M.A. (2014). Maternal choline supplementation programs greater activity of the phosphatidylethanolamine N-methyltransferase (PEMT) pathway in adult Ts65Dn trisomic mice. FASEB J..

[69] West A.A., Yan J., Jiang X., Perry C.A., Innis S.M., Caudill M.A. (2013). Choline intake influences phosphatidylcholine DHA enrichment in nonpregnant women but not in pregnant women in the third trimester. Am. J. Clin. Nutr..

[70] Taesuwan S., McDougall M.Q., Malysheva O.V., Bender E., Nevins J.E.H., Devapatla S., Vidavalur R., Caudill M.A., Klatt K.C. (2021). Choline metabolome response to prenatal choline supplementation across pregnancy: A randomized controlled trial. FASEB J..

[71] Li Z., Agellon L.B., Allen T.M., Umeda M., Jewell L., Mason A., Vance D.E. (2006). The ratio of phosphatidylcholine to phosphatidylethanolamine influences membrane integrity and steatohepatitis. Cell Metab..

[72] Dawaliby R., Trubbia C., Delporte C., Noyon C., Ruysschaert J.M., Van Antwerpen P., Govaerts C. (2016). Phosphatidylethanolamine Is a Key Regulator of Membrane Fluidity in Eukaryotic Cells. J. Biol. Chem..

[73] Van der Veen J.N., Lingrell S., da Silva R.P., Jacobs R.L., Vance D.E. (2014). The concentration of phosphatidylethanolamine in mitochondria can modulate ATP production and glucose metabolism in mice. Diabetes.

