# Supplementary material for: Prenatal Choline Supplement in a Maternal Obesity Model Modulates Offspring Hepatic Lipidomes
Source: Nutrients. 2023 Feb 15;15(4):965. doi: 10.3390/nu15040965 (PMC9963284; doi:10.3390/nu15040965)
Supplement: Supplementary file 1 [file nutrients-15-00965-s001.zip › nutrients-2176691-supplementary/Figure S1.pdf]

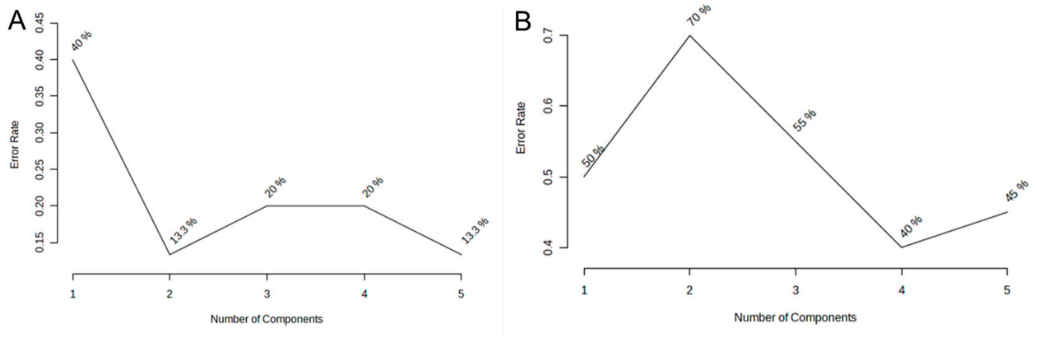

Supplementary Figure S1. Cross validation (CV) of Sparse partial least squares discriminant analysis (sPLS-DA) performance using 5-fold CV (A) at E17.5 and (B) after 6-week postweaning high-fat feedingof offspring.
