# Supplementary material for: Prenatal Choline Supplement in a Maternal Obesity Model Modulates Offspring Hepatic Lipidomes
Source: Nutrients. 2023 Feb 15;15(4):965. doi: 10.3390/nu15040965 (PMC9963284; doi:10.3390/nu15040965)
Supplement: Supplementary file 1 [file nutrients-15-00965-s001.zip › nutrients-2176691-supplementary/Figure S2.pdf]

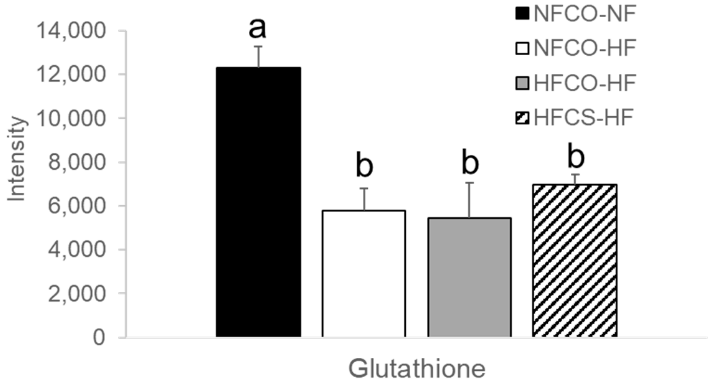

Supplementary Figure S2. Abundance of glutathione in the liver of male offspring mice after 6-week postweaning HF feeding. Glutathione was measured as one of the primary metabolites in the metabolomics panel using Gas chromatography Time-of-flight (GC-TOF) mass spectrometry (MS) at the West Coast Metabolomics Center (WCMC). Resulting peak intensities were normalized by tissue wet weight. n = 5-6/group; Values represent means ± SEM; analyzed with ANOVA and Post-hoc Fisher's LSD; a,b, any two groups with no overlapping letters were significantly different in their pair-wise comparison. CO, untreated control without choline supplement; CS, choline supplemented; HF, high fat; NF, normal fat.
