# Supplementary material for: Prenatal Choline Supplement in a Maternal Obesity Model Modulates Offspring Hepatic Lipidomes
Source: Nutrients. 2023 Feb 15;15(4):965. doi: 10.3390/nu15040965 (PMC9963284; doi:10.3390/nu15040965)
Supplement: Supplementary file 1 [file nutrients-15-00965-s001.zip › nutrients-2176691-supplementary/Figure S3.pdf]

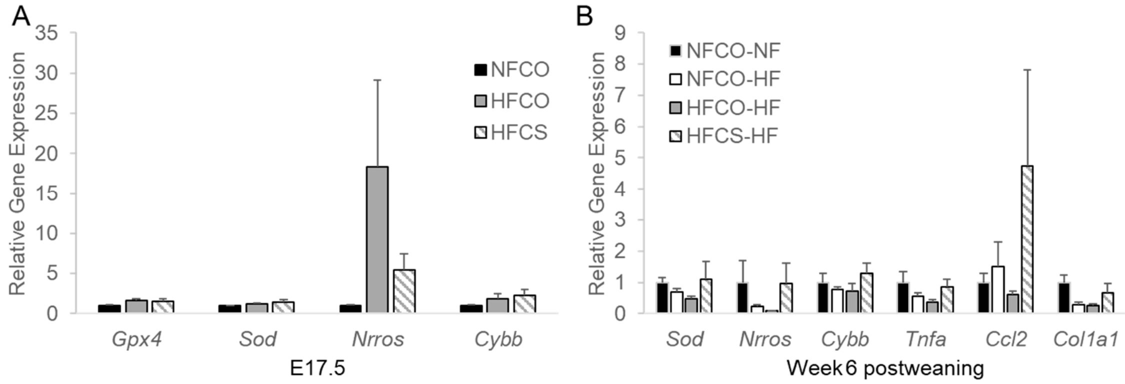

Supplementary Figure S3. Fetal and week 6 postweaning hepatic gene expression of proteins involved in oxidative stress regulation and inflammation. (A) E17.5; (B) week 6 postweaning; n = 6-10/group; Values represent means ± SEM; analyzed with ANOVA and Post-hoc Fisher's LSD; no significant difference between groups. CO, untreated control without choline supplement; CS, choline supplemented; HF, high fat; NF, normal fat. *Nrros*, negative regulator of reactive oxygen species; *Ccl2*, MCP-1/chemokine (C-C motif) ligand 2; *Col1a1*, COL1A1 collagen type I alpha 1 chain; *Cybb*, cytochrome b-245 beta; *Gpx4*, glutathione peroxidase 4; *Sod*, superoxide dismutase; *Tnfa*, tumor necrosis factor alpha.
