# Supplementary material for: Prenatal Choline Supplement in a Maternal Obesity Model Modulates Offspring Hepatic Lipidomes
Source: Nutrients. 2023 Feb 15;15(4):965. doi: 10.3390/nu15040965 (PMC9963284; doi:10.3390/nu15040965)
Supplement: Supplementary file 1 [file nutrients-15-00965-s001.zip › nutrients-2176691-supplementary/Supplemental Table S2.pdf]

Supplementary Table S2. Abundance of lipid species in the E17.5 fetal liver

Lipid species	HFCO [a]	HFCS [a]	FDR adjusted *p* value [b]
PC 32:1	0.25	0.33	0.005
PE 32:1	0.23	0.36	0.005
PE 38:1, PE O-40:8	1.29	4.01	0.005
PE O-38:6	1.82	4.05	0.026
PE 40:8, PE O-40:1	1.94	3.90	0.033
PE 42:7, PE O-42:0	3.13	3.37	0.033
PE O-40:5	1.02	3.28	0.033
PE O-42:5	1.30	3.69	0.033
PC O-42:11	0.28	0.36	0.043
PA O-42:6	3.13	3.16	0.051
PC 40:4	0.28	0.43	0.051
PE 34:1	0.29	0.80	0.051
PE O-42:10	1.19	3.35	0.051
PE O-34:2	1.43	3.48	0.066
PE O-40:6	2.51	3.43	0.066
PE 34:3	0.28	0.57	0.072
PE 34:4	0.29	0.64	0.072
PE O-42:8	1.49	3.45	0.072
PE 42:8, PE O-42:1	2.27	3.43	0.073
PS 36:4	0.76	0.30	0.073
PS 32:1	0.35	0.33	0.076
LPC 22:0	0.75	2.48	0.078
PC 32:2	0.29	0.54	0.082
PE O-40:4	1.46	3.31	0.090
PS O-40:5	3.37	2.04	0.090
PE O-42:11	1.75	3.32	0.104
PS 38:5	0.59	0.30	0.104
PE 42:9, PE O-42:2	1.63	3.29	0.104
PE 42:5	2.33	3.15	0.112
PC O-32:1	0.31	0.66	0.117
PE 40:1	1.25	3.03	0.117
PE 40:4	1.64	3.23	0.117
PE 40:5	0.45	1.40	0.117
PE O-42:6	1.91	3.21	0.121
PE 40:7, PE O-40:0	2.18	3.09	0.134
PE O-42:9	1.48	3.10	0.137
PG 34:1	0.52	0.32	0.137
PE 42:10, PE O-42:3	1.20	2.91	0.142
PI 40:5	0.32	0.52	0.142

PA O-42:5	3.02	1.42	0.142
PC 38:0, PC O-40:7	0.54	0.32	0.142
PS 36:2	3.09	1.90	0.142
PE O-40:3	2.09	3.00	0.153
PS 42:11, PS O-42:4	1.89	3.05	0.153
PA 36:4	0.36	0.42	0.153
PC 40:8, PC O-40:1	2.82	2.34	0.153
PE 36:1	0.82	2.33	0.153
PE O-34:6	2.44	2.76	0.153
PE O-42:7	1.85	3.04	0.153
LPI 18:0	1.03	0.39	0.163
PC 36:6	0.76	2.18	0.165
PE 36:0, PE O-38:7	0.91	2.44	0.165
PS 38:4	1.12	0.41	0.165
LPI 20:4	0.99	0.39	0.166
LPC 22:5	0.51	0.34	0.171
PC O-34:2	2.24	2.75	0.183
PE 32:2	0.35	0.55	0.194
PI 36:5	0.42	0.40	0.225
PS 38:3	1.39	0.50	0.225
PC 30:1	0.36	0.61	0.230
PC O-38:5	1.15	0.45	0.241
PS 38:6	1.21	0.46	0.241
PG 34:0, PG O-36:7	0.61	0.36	0.249
LPE 16:1	0.54	0.37	0.262
PE 40:0	1.72	2.73	0.262
PE 36:2	1.10	2.44	0.302
PE O-32:0	0.90	2.20	0.303
PE 32:0	0.39	0.72	0.321
PG 36:5	0.45	0.42	0.321
PI 34:0, PI O-36:7	0.89	0.41	0.321
PC O-36:5	1.17	2.46	0.322
PC O-38:4	1.87	0.73	0.323
PE O-32:1	0.39	0.74	0.325
PC 38:1, PC O-40:8	0.52	1.31	0.338
PC O-34:5	1.57	2.57	0.338
PE O-34:1	0.42	0.89	0.338
PE O-36:3	1.11	2.37	0.338
PS 40:6	1.22	0.50	0.342
LPC 16:1	0.59	0.40	0.346
PS 34:2	2.01	0.83	0.346
PC 34:0, PC O-36:7	0.40	0.65	0.357
PC 36:0, PC O-38:7	0.42	0.50	0.357

PE 42:6	1.71	2.50	0.357
LPC O-22:6	0.62	0.40	0.359
PE 38:2, PE O-40:9	1.08	2.29	0.359
PC O-30:3	1.25	0.52	0.377
PE 34:0, PE O-36:7	0.57	1.38	0.377
PI 38:3	0.45	0.48	0.384
PS 36:3	1.65	0.68	0.384
PE 42:1	0.65	1.58	0.397
PI 38:2, PI O-40:9	0.44	0.49	0.397
PC O-38:6	0.43	0.83	0.398
PA 40:6	1.81	2.33	0.408
PC O-38:3	2.33	1.30	0.408
PE O-36:1	0.71	1.69	0.408
PG 36:1	0.60	0.42	0.408
PG 36:2	2.39	1.46	0.408
PS 38:0, PS O-40:7	1.80	2.34	0.408
PS 40:7, PS O-40:0	0.62	0.42	0.408
LPC 18:1	0.70	0.43	0.409
LPC 20:3	0.84	0.45	0.409
LPC 22:6	1.17	0.52	0.409
LPE 22:3	0.64	0.43	0.409
PC O-34:6	1.45	2.34	0.409
PE O-36:6	1.73	2.31	0.409
PG 38:5	2.06	2.11	0.409
PS 34:1	0.59	0.43	0.409
PS 40:4	0.45	0.50	0.409
PS O-36:2	2.33	1.67	0.409
PS O-42:11	0.45	0.50	0.409
PE 40:9, PE O-40:2	1.89	2.20	0.422
PE O-38:2	1.33	2.28	0.422
PE 40:3	1.07	2.12	0.424
PS O-38:4	1.51	0.66	0.424
LPC 20:4	1.37	0.61	0.436
PE 36:3	1.41	2.27	0.436
PE 42:4	1.84	2.18	0.437
PE 38:0, PE O-40:7	0.89	1.89	0.450
LPC 20:5	0.96	0.49	0.459
PE 42:3	1.86	2.12	0.459
PC 34:4	0.45	0.72	0.461
PE O-38:1	1.12	2.09	0.468
PG 34:4	0.56	0.47	0.474
LPC 18:3	0.95	0.50	0.501
LPC 22:4	0.92	0.50	0.501

LPE 20:4	1.31	0.62	0.501
LPE O-18:0	1.91	1.99	0.501
PC O-32:5	0.85	1.76	0.501
PE 40:6	1.52	2.14	0.501
PI 36:4	0.57	0.48	0.501
PS 40:8, PS O-40:1	1.28	0.60	0.501
PS 42:7, PS O-42:0	0.47	0.65	0.501
PS O-40:6	1.56	2.16	0.501
PC 42:7, PC O-42:0	1.70	2.07	0.515
LPE 18:1	0.82	0.49	0.526
PC O-40:6	0.63	0.48	0.526
PI 36:1	0.55	0.51	0.526
PC O-36:4	1.43	0.68	0.539
PC O-40:5	1.41	2.06	0.561
PS 36:5	0.95	0.52	0.561
PE 30:0	0.50	0.80	0.571
LPC 18:2	1.69	0.86	0.578
PC 38:3	1.94	1.69	0.599
PC 40:1	0.97	1.80	0.599
PC O-36:3	2.00	1.33	0.599
LPC 22:3	0.84	0.52	0.605
LPE 18:2	1.88	1.10	0.605
PC 34:2	1.88	1.73	0.605
PE 40:2	1.06	1.86	0.605
PI 34:1	0.66	0.51	0.605
PS O-42:5	1.99	1.40	0.605
PC O-30:0	1.08	1.87	0.607
PI 36:3	0.59	0.54	0.621
PE 36:5	0.56	0.56	0.625
LPE O-22:0	1.93	1.33	0.638
LPS 18:1	0.94	0.55	0.641
PC 36:2	1.67	1.84	0.641
PE O-38:5	0.54	0.85	0.665
LPE 22:5	0.83	0.54	0.666
LPE 22:0	1.35	1.88	0.675
PC O-42:6	0.61	1.10	0.675
PE 32:3	0.75	1.42	0.675
LPC O-22:0	0.54	0.65	0.681
LPC 16:0	0.93	0.56	0.683
PC 42:10, PC O-42:3	1.50	0.82	0.695
PC O-32:0	0.90	1.60	0.695
PE 34:2	0.60	1.03	0.695
PE O-38:3	0.95	1.65	0.695

LPE 18:0	0.75	0.55	0.701
PS O-40:3	1.55	1.78	0.707
PC 30:0	0.91	1.60	0.707
LPC O-18:1	0.94	0.59	0.709
PC 42:6	1.79	1.44	0.709
PE 36:6	0.82	1.47	0.709
PE O-36:0	0.60	0.60	0.709
PG 34:2	1.40	0.78	0.709
PI 38:5	0.63	0.57	0.709
PI 40:4	0.56	0.80	0.709
PS 42:2	1.16	1.76	0.709
PC 42:0	0.84	1.47	0.713
LPS 18:0	1.01	0.61	0.719
LPE 20:3	0.96	0.60	0.731
PC O-36:0	0.60	0.96	0.732
LPC 18:0	1.09	0.65	0.743
PC O-40:4	0.65	0.60	0.743
PE O-36:2	1.01	1.63	0.743
PI 38:6	0.58	0.74	0.743
PC 38:4	1.70	1.47	0.743
PC 34:1	0.59	0.71	0.751
PE 30:1	0.69	1.18	0.751
PI O-42:11	0.58	0.80	0.751
PC O-42:7	0.80	1.36	0.757
PC 40:3	0.67	1.07	0.801
PC 36:5	0.70	0.62	0.819
LPE 22:6	1.50	0.96	0.823
PA 40:7, PA O-40:0	1.60	1.45	0.823
PS 42:9, PS O-42:2	1.43	0.90	0.829
PC 36:3	1.54	1.46	0.855
PE O-38:4	1.52	1.49	0.855
LPC O-18:0	1.41	0.90	0.858
PC 42:9, PC O-42:2	0.66	0.68	0.858
PC O-42:10	0.65	0.69	0.858
PC 32:0	0.74	1.16	0.858
PC 40:5	0.71	0.65	0.864
PE 38:7	1.25	1.57	0.864
PG 40:6	1.12	0.72	0.864
PC O-38:2	1.56	1.25	0.866
PE 38:6	1.39	1.53	0.866
PI 40:6	0.72	1.09	0.873
PC 32:3	0.80	1.24	0.876
PC 36:1	0.68	0.97	0.876

PS 40:5	1.09	0.73	0.897
PC O-42:8	0.87	1.31	0.901
PS O-36:1	1.44	1.00	0.901
PC O-34:1	0.66	0.83	0.902
PG 36:4	0.82	1.23	0.909
PS 42:0	1.00	1.41	0.911
PC 42:1	0.97	1.38	0.922
PG 34:3	0.76	1.10	0.922
PS 42:6	1.00	1.40	0.925
PI 34:2	1.19	0.82	0.931
PC 40:0	1.05	1.41	0.941
PC 40:6	1.18	0.82	0.941
PC O-36:6	0.78	1.12	0.941
PI O-38:4	0.71	0.75	0.941
PE 42:2	1.36	1.00	0.958
PC 38:2, PC O-40:9	0.75	1.01	0.959
PC 36:4	1.40	1.17	0.967
LPE 16:0	1.16	0.84	0.985
LPE 18:3	1.36	1.22	0.986
PA 38:4	1.30	1.28	0.986
PC 34:6	1.33	1.13	0.986
PC 38:5	0.83	0.75	0.986
PC O-36:1	0.99	1.29	0.986
PC O-42:9	1.00	1.28	0.986
PE 36:4	0.75	0.85	0.986
PE 38:4	0.94	1.23	0.986
PE O-34:0	0.98	1.30	0.986
PG 40:7, PG O-40:0	1.20	0.91	0.986
PG 40:9, PG O-40:2	0.76	0.97	0.986
PI 36:2	0.75	0.90	0.986
PI 38:4	1.02	0.80	0.986
PS 42:3	1.28	0.97	0.986
PS 42:5	1.19	1.32	0.986
PC 40:7, PC O-40:0	1.19	0.93	0.991
PC 42:3	1.25	1.02	0.991
PE 38:3	0.89	1.15	0.991
PE 38:5	0.87	1.14	0.991
PE O-36:4	0.84	1.07	0.991
PS 32:0	0.88	0.78	0.991
PS 38:7	1.28	1.04	0.991
PA 36:5	0.83	0.84	0.992
PA 38:5	0.94	0.81	0.992
PC 34:3	1.05	0.88	0.992

PC 38:6	1.16	0.92	0.992
PC 40:2	1.08	1.22	0.992
PC 42:2	1.03	1.19	0.992
PC 42:8, PC O-42:1	1.11	1.24	0.992
PC O-34:0	0.84	1.03	0.992
PG 42:9, PG O-42:2	0.89	0.83	0.992
PS 40:0	1.00	1.21	0.992
PS 40:1	0.85	0.99	0.992
PS 40:3	1.15	0.91	0.992
PS 42:8, PS O-42:1	1.22	1.09	0.992
PS O-42:10	1.17	0.95	0.992
PS O-42:7	0.98	1.17	0.992
PS O-42:8	0.83	1.00	0.992
PS 34:0, PS O-36:7	1.12	0.94	0.994
LPC O-16:0	1.08	0.91	1.000
PS 42:1	0.96	1.12	1.000
PC 38:7	1.10	0.97	1.000
PC 42:4	0.98	1.00	1.000
PC 42:5	1.07	1.04	1.000
PC O-36:2	1.09	1.11	1.000
PG 36:3	0.95	1.01	1.000
PG 38:4	0.86	0.90	1.000
PG 38:6	1.14	1.08	1.000
PG 38:7	0.96	0.91	1.000
PG 40:8, PG O-40:1	0.92	0.97	1.000
PS 36:0, PS O-38:7	1.01	1.00	1.000
PS 36:1	0.95	0.95	1.000
PS 38:1, PS O-40:8	0.96	0.91	1.000
PS 38:2, PS O-40:9	1.03	1.04	1.000
PS 40:2	0.90	0.88	1.000
PS 42:4	1.14	1.01	1.000
PS O-42:9	0.90	0.95	1.000

[a] Values are fold changes compared to the control group (NFCO).

[b]P values are analyzed by ANOVA across different groups and adjusted for FDR using MetaboAnalyst 5.0.

NF, normal fat; CO, no choline supplement; HF, high fat; CS, choline supplement. FDR: false discovery rate
