# Supplementary material for: Prenatal Choline Supplement in a Maternal Obesity Model Modulates Offspring Hepatic Lipidomes
Source: Nutrients. 2023 Feb 15;15(4):965. doi: 10.3390/nu15040965 (PMC9963284; doi:10.3390/nu15040965)
Supplement: Supplementary file 1 [file nutrients-15-00965-s001.zip › nutrients-2176691-supplementary/Supplemental Table S3.pdf]

Supplemental Table S3. Abundance of lipid species in male offspring livers after 6-week post-weaning high-fat feeding.

Lipid species	NFCO-HF[a]	HFCO-HF[a]	HFCS-HF[a]	FDR adjusted *p* value [b]
GlcCer d42:1	0.24	0.23	0.44	0.002
GlcCer d42:2	0.25	0.22	0.41	0.002
PG 40:6 | PG 18:1_22:5	0.26	0.23	0.59	0.002
TG 56:4 | TG 18:0_18:2_20:2	4.46	3.96	3.35	0.002
PE 38:7 | PE 16:1_22:6	0.23	0.31	0.24	0.002
PG 36:2 | PG 18:1_18:1	0.25	0.23	0.30	0.002
TG 56:8 Isomer A	4.20	4.24	2.78	0.002
PE 38:5 | PE 18:0_20:5	0.28	0.24	0.65	0.003
FA 24:1 (nervonic acid)	0.24	0.25	0.45	0.003
PC 33:3;O | PC 18:2_15:1;O	3.94	3.46	3.99	0.003
PE 38:7	0.25	0.30	0.25	0.004
SM d37:1	3.65	4.26	3.24	0.004
TG 58:10	3.92	4.21	2.78	0.004
PE 36:5 | PE 16:1_20:4	0.26	0.26	0.29	0.004
PE P-34:2 or PE O-34:3	0.28	0.25	0.57	0.004
TG 56:9	4.06	3.98	2.82	0.004
CL 70:7 | CL 16:1_18:2_18:2_18:2	0.27	0.27	0.27	0.005
GlcCer d41:1	0.29	0.28	0.72	0.006
PE 34:3 | PE 16:1_18:2	0.26	0.27	0.30	0.007
SM d36:0	4.17	3.37	1.83	0.007
TG 54:7 Isomer A	3.85	3.99	2.33	0.007
PC 32:0	0.37	0.22	0.56	0.007
PE 32:1 | PE 16:0_16:1	0.27	0.29	0.27	0.008
TG 53:3;2O | TG 16:0_18:2_19:1;2O	3.75	3.68	1.75	0.008
LPC 16:1	0.26	0.31	0.27	0.008
PC 30:0	0.32	0.23	0.36	0.008
PI 38:5	0.28	0.26	0.53	0.008
TG 56:8 Isomer B	3.77	3.77	1.93	0.008
CL 72:6 | CL 18:1_18:2_18:1_18:2	0.25	0.29	0.35	0.009
PC 38:5 Isomer B	0.32	0.25	0.59	0.009
SM d42:2 Isomer A	0.31	0.24	0.48	0.009
TG 52:5	3.66	3.80	1.95	0.009
TG 54:8	3.68	3.83	2.86	0.009
PE O-38:6 | PE O-16:1_22:5	0.38	0.27	0.85	0.010
PC 32:1	0.31	0.29	0.27	0.011
PI 40:4	0.33	0.26	0.29	0.011

TG 60:10 I TG 18:0_20:4_22:6	3.79	3.66	2.18	0.011
FA 14:1 (physeteric acid)	0.27	0.35	0.74	0.013
PC 36:5 Isomer C	0.31	0.28	0.29	0.013
PG 36:3 I PG 18:1_18:2	0.26	0.30	0.51	0.013
TG 54:6 I TG 18:2_18:2_18:2	3.82	3.29	1.79	0.014
TG 52:6	3.74	3.53	2.08	0.014
SM d38:1	3.39	3.84	2.07	0.015
TG 54:6 Isomer C	3.98	3.23	2.19	0.016
PC 37:4	3.24	2.79	3.86	0.017
PE 40:5 I PE 18:0_22:5	0.28	0.32	0.67	0.020
CE 18:2	2.81	3.49	3.52	0.021
TG 56:7 Isomer A	3.93	3.12	2.17	0.021
PC 36:3 Isomer A	0.26	0.31	0.38	0.021
Cer d38:1	2.60	4.20	2.25	0.021
PC 38:4 Isomer C	3.15	3.04	3.65	0.021
PG 38:4 I PG 18:0_20:4	0.41	0.53	1.51	0.021
PC 39:4	2.63	2.52	4.13	0.021
SM d42:2	0.34	0.25	0.50	0.021
PC 40:4 I PC 20:0_20:4	3.28	3.18	3.37	0.022
PC P-34:0 or PC O-34:1	0.34	0.26	0.58	0.022
PE P-40:4 or PE O-40:5 Isomer A	2.72	2.60	4.04	0.022
SM d40:2 Isomer A	0.33	0.31	0.28	0.022
TG 52:4	3.44	3.08	1.47	0.022
PC 31:0	0.33	0.28	0.61	0.022
PC 38:7	0.37	0.37	0.25	0.022
PC 39:6	3.08	3.77	2.64	0.022
PG 34:1 I PG 16:0_18:1	0.29	0.33	0.32	0.022
TG 54:7 I TG 16:0_18:2_20:5	3.60	3.10	1.68	0.022
TG 55:4;2O I TG 18:1_18:2_19:1;2O	3.50	3.39	1.88	0.022
PC 36:5 I PC 16:1_20:4	0.32	0.30	0.31	0.022
PC 40:4 Isomer B	3.30	3.10	3.29	0.022
TG 56:10	3.20	3.71	2.39	0.022
PC 34:3 Isomer A	0.32	0.43	0.27	0.024
Cer d40:1	2.37	4.03	1.73	0.024
LPC 18:1	0.28	0.30	0.44	0.024
Cer 40:1;2O I Cer 18:1;2O/22:0	2.40	4.01	1.76	0.025
DG 36:4 Isomer A	2.61	3.93	2.58	0.025
DG 36:5	2.62	3.62	3.12	0.025
PC 35:2	2.97	3.55	2.98	0.025
PE O-38:5 I PE O-16:1_22:4	0.35	0.26	0.55	0.025
TG 51:4	3.60	2.93	1.65	0.025

PI 40:6 l PI 18:0_22:6	0.50	0.66	0.25	0.026
TG 55:5 l TG 17:0_18:1_20:4	3.78	2.83	1.98	0.029
TG 56:5 l TG 18:0_18:1_20:4	3.64	2.94	1.82	0.029
Cer 38:1;2O l Cer 18:1;2O/20:0	2.25	4.02	1.91	0.030
TG 54:6 l TG 16:0_18:2_20:4	3.82	2.76	2.05	0.030
TG 55:7 l TG 15:0_18:1_22:6	3.72	2.90	2.00	0.030
PE 36:4 Isomer B	0.27	0.37	0.36	0.030
TG 53:5	3.57	2.84	1.65	0.031
Cer d40:2	2.59	3.89	2.19	0.031
PE O-40:5 l PE O-18:1_22:4	2.54	2.22	3.91	0.031
PE P-40:7 or PE O-40:8	0.33	0.30	0.34	0.032
SM d38:2	3.45	3.09	2.66	0.032
TG 60:11	3.52	3.09	2.13	0.033
PE 40:8 l PE 18:2_22:6	0.32	0.60	0.31	0.033
SM d40:0	2.50	2.70	0.96	0.033
Cer 39:1;2O l Cer 17:1;2O/22:0	2.67	3.75	2.44	0.034
Cer d39:1	2.57	3.74	2.54	0.035
PI 36:3 l PI 18:1_18:2	0.28	0.38	0.65	0.036
PC 38:6 Isomer C	0.37	0.36	0.28	0.037
TG 54:6 Isomer B	3.16	2.97	1.47	0.038
Cer 40:2;2O l Cer 18:2;2O/22:0	2.53	3.70	1.78	0.040
PC 36:5 Isomer A_1	1.94	3.90	1.96	0.040
PC 35:2 Isomer B	2.68	3.26	3.08	0.040
PE P-38:4 or PE O-38:5	2.39	1.91	3.79	0.041
PE 38:5 l PE 18:1_20:4	0.29	0.41	0.73	0.042
TG 58:10 l TG 16:0_20:4_22:6	3.52	2.82	1.86	0.042
PG 44:11 l PG 22:5_22:6	0.37	0.41	1.04	0.043
DMPE 32:0 l DMPE 16:0_16:0	0.42	0.30	0.76	0.044
PC 32:3	0.31	0.34	0.36	0.044
PE P-38:6 or PE O-38:7	0.39	0.28	0.37	0.044
PC 38:5 Isomer B_1	0.41	0.29	0.71	0.046
SM d40:1	2.32	3.64	1.61	0.046
CL 74:8 l CL 36:4_38:4	1.16	3.21	2.19	0.047
CL 74:8 l CL 18:1_18:1_18:2_20:4	1.08	2.93	2.37	0.048
PE 36:1	0.33	0.36	0.32	0.048
SM d42:2 Isomer B	0.34	0.32	0.62	0.048
PC O-34:0	0.80	0.89	2.60	0.049
CL 72:8 l CL 18:2_18:2_18:2_18:2	2.68	3.53	2.26	0.050
PC 38:4 Isomer A	0.42	0.30	0.34	0.050
LPC 16:0	0.28	0.44	0.37	0.051
PC 16:0/9:0 CHO	1.27	0.56	0.39	0.053

TG 60:4	3.23	3.03	2.05	0.053
PE P-36:2 or PE O-36:3	0.43	0.61	1.45	0.053
LPE 16:0	0.27	0.46	0.48	0.054
PG 38:6 | PG 18:2_20:4	1.82	2.10	3.69	0.055
LPI 20:4	0.30	0.78	0.83	0.055
PC 33:1	0.35	0.36	0.33	0.058
TG 60:12 | TG 16:0_22:6_22:6	3.29	2.79	1.77	0.058
PC P-38:4 or PC O-38:5 Isomer B	2.45	1.78	3.51	0.058
PC 36:3 Isomer B	0.42	0.38	0.29	0.059
PE P-40:4 or PE O-40:5 Isomer B	2.66	2.33	3.38	0.061
SM d42:3	0.60	0.33	1.03	0.062
SM d43:1	0.36	0.32	0.60	0.063
PC P-38:5 or PC O-38:6 Isomer A	0.30	0.39	0.38	0.064
CoQ10	0.31	0.35	0.51	0.068
TG 56:9 | TG 16:1_18:2_22:6	3.10	2.65	1.47	0.068
TG 54:5 | TG 18:1_18:2_18:2	3.12	2.61	1.50	0.073
PC 34:1	0.36	0.40	0.32	0.074
DMPE 36:1 | DMPE 18:0_18:1	0.32	0.35	0.47	0.077
PC 36:5 Isomer B	0.41	0.35	0.33	0.077
TG 53:4	3.08	2.28	1.30	0.082
PC 36:5 Isomer A	1.64	3.47	1.68	0.085
PC 36:1	0.34	0.34	0.47	0.090
PG 36:2 | PG 16:0_20:2	1.40	1.88	3.31	0.093
LPC 20:1	0.35	0.34	0.55	0.095
LPC 20:3	0.39	0.34	0.37	0.095
PC 34:0	1.88	1.15	3.02	0.095
PC 40:5 Isomer B	0.41	0.34	0.76	0.095
PE 38:5	0.37	0.46	0.95	0.095
PE 40:7 | PE 18:1_22:6	0.31	0.58	0.40	0.095
SM d39:1	2.46	3.19	2.35	0.095
TG 42:1	0.40	0.41	0.32	0.095
TG 54:5 Isomer A	3.07	2.40	1.45	0.095
TG 58:3	2.40	2.66	1.14	0.095
PI 38:3 | PI 18:0_20:3	0.55	0.51	0.29	0.095
PC 32:2	0.43	0.36	0.34	0.095
DG 36:4 Isomer B	2.15	3.08	2.68	0.096
PC 37:6	2.28	3.15	1.49	0.098
TG 54:5 Isomer B	3.04	2.32	1.39	0.098
SM d40:2 Isomer B	2.42	3.20	1.73	0.099
PC O-32:0	0.41	0.34	0.71	0.101
PE 36:4	0.32	0.53	0.40	0.101

PE 37:2 | PE 19:0_18:2	1.96	2.65	3.07	0.101
TG 51:3	2.84	2.12	1.15	0.103
TG O-52:1 | TG O-18:0_16:0_18:1	1.34	2.07	3.09	0.106
PG 38:5 | PG 18:1_20:4	0.50	0.53	1.38	0.108
LPE 18:1	0.32	0.46	0.67	0.112
DG 38:6	2.12	2.77	2.87	0.116
CL 72:7 | CL 18:1_18:2_18:2_18:2	0.31	0.49	0.44	0.119
PC 36:5 Isomer D	0.45	0.37	0.35	0.123
PC P-38:3 or PC O-38:4 Isomer B	2.78	2.39	2.70	0.123
LPE 20:4	0.31	0.57	0.55	0.125
TG 52:3	2.41	2.02	0.93	0.126
PE P-40:4 or PE O-40:5	2.07	1.83	3.20	0.134
SM d41:2 Isomer A	0.48	0.35	0.82	0.134
TG 56:6	2.93	2.26	1.42	0.134
LPE 22:6	0.31	0.65	0.60	0.136
PC 36:2	2.46	2.78	2.50	0.140
PE 38:2 | PE 20:0_18:2	2.18	2.93	2.46	0.141
SM d36:1	2.36	1.75	3.02	0.141
PC 34:3 | PC 16:0_18:3	1.80	2.50	2.91	0.143
SM d41:2	0.51	0.46	1.18	0.143
TG 53:3	2.71	1.90	1.09	0.143
PI 39:4 | PI 19:0_20:4	1.47	1.69	3.10	0.151
PE 34:1_1	0.34	0.41	0.57	0.152
PC 38:4 Isomer B	0.57	0.33	0.75	0.152
LPC 20:3_1	0.36	0.46	0.38	0.152
PC 40:7	0.59	0.45	0.33	0.156
PC 37:2	2.13	2.21	2.99	0.157
PI 37:4 | PI 17:0_20:4	1.57	2.00	3.05	0.158
TG 72:4 | TG 18:1_36:1_18:2	2.98	2.27	1.95	0.160
PE P-40:5 or PE O-40:6	2.45	1.89	2.86	0.163
PC 42:5 Isomer A	2.78	1.72	1.13	0.164
PE 36:3	0.40	0.49	0.93	0.171
TG O-50:1 | TG O-16:0_16:0_18:1	0.42	0.43	0.36	0.171
PC 33:0	1.17	0.88	2.44	0.175
PC 38:6 Isomer B	1.55	1.90	0.68	0.178
TG 55:5 | TG 18:1_18:2_19:2	2.79	2.01	1.32	0.189
TG 56:7 Isomer B	2.46	2.39	1.29	0.195
PC 38:6	1.33	1.80	0.63	0.197
PE 40:7	0.36	0.75	0.52	0.198
PC 40:6 Isomer A	1.74	1.45	2.94	0.198
LPC 14:0	0.43	0.40	0.40	0.200

DG 40:8 | DG 18:2_22:6	1.75	2.83	2.22	0.203
PC 33:2	1.97	2.95	1.80	0.203
PE 37:4 | PE 17:0_20:4	2.08	2.72	2.37	0.203
FA 16:1 (palmitoleic acid)	0.36	0.45	0.48	0.207
CAR 16:0	2.30	2.10	2.72	0.209
LPI 18:0	0.42	1.12	0.92	0.209
PC 42:6 Isomer B	2.46	2.54	1.51	0.209
Cer 40:0;2O | Cer 18:0;2O/22:0	2.12	2.50	1.20	0.211
PC 41:6 | PC 19:0_22:6	2.22	2.48	2.49	0.217
PC 37:3	0.49	0.35	0.52	0.221
PC 42:7	0.57	0.40	0.39	0.221
PE 36:2	1.87	2.73	2.27	0.224
PG 36:4 | PG 18:2_18:2	0.97	1.52	2.45	0.225
TG 54:2 | TG O-19:1_17:0_18:1	2.02	1.90	2.84	0.225
LPC 18:3	0.63	1.28	1.76	0.225
TG 59:2	1.13	0.82	0.43	0.230
SM d34:0	2.55	2.44	1.76	0.234
PE P-36:4 or PE O-36:5	0.61	0.43	1.08	0.234
PC 42:10	0.80	0.56	0.37	0.238
PE 39:6 | PE 17:0_22:6	1.80	2.67	2.25	0.243
TG 60:6	2.68	2.20	1.98	0.243
PC 34:3 Isomer C	1.62	2.28	2.61	0.243
TG 56:5 Isomer B	2.66	1.94	1.33	0.243
PC 38:5 Isomer A	0.53	0.37	0.73	0.251
TG 46:2	1.05	0.71	0.42	0.253
TG 53:2	2.13	1.36	0.81	0.266
PC P-36:1 or PC O-36:2 A	1.32	1.56	2.71	0.267
PC 42:10 | PC 20:4_22:6	0.82	0.60	0.38	0.271
TG 49:0	1.71	0.84	0.63	0.273
PC 35:2 Isomer A	0.40	0.44	0.62	0.276
PC 40:7 Isomer A	0.66	0.45	0.40	0.277
PC 37:5 Isomer B	1.01	0.56	1.53	0.281
PE 34:1	0.40	0.49	0.74	0.285
PS 36:4	0.47	0.46	0.40	0.287
TG 50:4	2.06	1.47	0.82	0.287
PC P-40:3 or PC O-40:4	1.63	1.50	2.71	0.294
TG 51:1	2.22	1.39	0.89	0.296
PC 40:8	1.06	0.83	0.44	0.298
SM d36:2	1.28	0.88	2.21	0.301
GlcCer d40:1	0.40	0.55	0.46	0.302
Cer 42:2;2O | Cer 18:1;2O/24:1	0.39	0.49	0.53	0.302

PI 40:4 ǀ PI 20:0_20:4	1.51	2.09	2.52	0.302
TG 52:2 ǀ TG 16:0_18:1_18:1	1.36	1.14	0.54	0.306
PC 38:2 ǀ PC 18:0_20:2	2.27	2.30	2.09	0.322
LPC 22:6	0.39	0.67	0.53	0.326
PE 40:8	0.54	1.16	0.55	0.326
TG 49:1	1.76	1.04	0.67	0.335
TG 51:2	1.86	1.33	0.74	0.340
FA 14:0 (myristic acid)	0.42	0.49	0.72	0.346
TG 53:1	2.29	1.44	1.01	0.351
PE P-36:1 or PE O-36:2	0.75	0.48	1.17	0.352
PG 42:10 ǀ PG 20:4_22:6	1.34	1.81	2.47	0.352
PC 36:6	0.72	0.56	0.40	0.355
FA 18:1 (oleic acid)	0.39	0.64	0.73	0.359
LPC 20:0_1	1.90	2.23	2.27	0.359
PC 38:6 ǀ PC 18:2_20:4	2.34	1.54	2.14	0.359
PC 42:5 Isomer B	0.71	0.39	0.62	0.359
PE 40:6 ǀ PE 18:0_22:6	0.48	0.75	0.44	0.359
TG 49:2	1.81	1.19	0.72	0.359
TG 51:0 ǀ TG 17:0_17:0_17:0	2.24	0.98	1.19	0.359
TG 54:2	1.62	0.88	0.64	0.359
PS 36:4 ǀ PS 16:0_20:4	0.52	0.52	0.41	0.367
PE 37:4	1.59	2.24	2.25	0.368
PE 38:3 ǀ PE 18:0_20:3	0.56	0.90	0.45	0.377
PE 38:2	1.81	2.38	2.02	0.383
LPE 18:2	0.44	0.83	0.96	0.384
PE 38:6	0.54	1.17	0.60	0.388
FA 20:2 (eicosadienoic acid)	0.90	1.29	2.08	0.392
TG 54:1	1.78	1.28	0.74	0.392
TG 48:2	0.85	0.57	0.44	0.392
DG 34:2	1.75	2.46	1.51	0.395
PE 38:6 ǀ PE 18:2_20:4	0.99	1.26	2.19	0.395
TG 46:1	0.88	0.56	0.45	0.395
TG 50:2	1.21	0.85	0.51	0.395
TG 56:3	1.71	0.96	0.70	0.395
PE 40:4 ǀ PE 18:0_22:4	0.94	1.17	2.12	0.402
TG 49:3	1.82	1.23	0.76	0.402
Cer d34:0	1.53	2.40	1.28	0.403
LPC 20:4	0.41	0.62	0.73	0.403
TG 48:1	0.97	0.64	0.46	0.403
TG 56:5 Isomer A	2.21	1.73	1.14	0.403
TG 58:6	2.42	1.68	1.40	0.403

TG O-53:5 | TG O-19:2_16:0_18:3	1.42	2.14	0.98	0.403
LPC 22:6_1	0.43	0.82	0.62	0.403
TG 50:5	2.25	1.71	1.17	0.404
TG 52:1	1.71	0.99	0.71	0.407
PE 40:4 | PE 20:0_20:4	1.38	2.36	1.76	0.410
FA 18:3 (linolenic acid)	1.01	1.82	1.94	0.411
PC 39:7	1.68	1.23	0.70	0.411
TG 55:1 | TG 16:0_21:0_18:1	1.93	1.50	0.88	0.411
PC 34:4	0.68	0.41	0.67	0.415
TG 50:3 Isomer A	1.48	1.05	0.62	0.421
TG 54:4	1.98	1.41	0.90	0.423
FA 17:0 (margaric acid)	1.10	1.60	2.18	0.423
FA 18:2 (linoleic acid)	1.14	1.94	2.00	0.423
PC P-36:4 or PC O-36:5	1.03	0.86	1.94	0.423
TG 55:4 | TG 18:1_19:1_18:2	2.08	1.30	0.95	0.423
TG 57:1	1.10	0.87	0.50	0.423
CAR 18:2	2.01	2.05	2.05	0.427
FA 22:2 (docosadienoic acid)	0.77	1.21	1.78	0.434
PC 38:6 Isomer A	2.25	1.49	1.92	0.434
PC 40:6 Isomer B	1.81	1.97	1.05	0.434
TG 52:5 | TG 16:1_18:2_18:2	1.66	1.50	0.78	0.434
TG 54:3	1.42	1.01	0.60	0.434
TG 56:1	1.28	1.10	0.57	0.434
TG 56:2	1.51	1.14	0.65	0.434
PE 36:3 | PE 18:1_18:2	0.62	0.82	1.44	0.434
TG 50:1	0.89	0.61	0.46	0.435
PI 36:4 | PI 16:0_20:4	0.43	0.58	0.64	0.441
Cer d43:1	0.58	0.51	0.99	0.444
PC 38:2	2.21	1.14	1.46	0.444
TG 44:0	0.82	0.48	0.53	0.444
DG 36:3	1.59	2.34	1.57	0.446
PE 38:6 Isomer B	0.59	1.31	0.76	0.446
TG 58:7 | TG 18:1_18:1_22:5	2.24	1.72	1.33	0.449
LPC 20:0	1.87	2.17	1.89	0.449
LPC 18:2	0.43	0.66	0.72	0.452
PE 40:4	0.93	1.15	1.99	0.452
PE 38:3	0.62	0.95	0.49	0.457
PG 40:8 | PG 18:2_22:6	0.65	1.31	1.38	0.457
SM d41:2 Isomer B	0.90	0.63	1.45	0.458
TG 58:2	1.29	1.07	0.58	0.458
PC 40:7 Isomer B	0.72	0.57	0.45	0.460

PC P-34:1 or PC O-34:2	1.01	0.70	1.60	0.468
SM d42:1	0.48	0.50	0.56	0.468
TG 55:2	1.81	1.18	0.82	0.468
TG 58:1	0.93	0.78	0.47	0.468
TG 60:2 | TG 24:0_18:1_18:1	1.01	0.73	0.49	0.468
PC 39:6 | PC 17:0_22:6	1.21	0.69	0.58	0.471
PC 40:4 Isomer A	1.47	0.98	1.96	0.481
GlcCer d38:1	0.44	0.62	0.67	0.482
LPC P-18:0 or LPC O-18:1	0.54	0.62	1.04	0.482
CE 22:4	0.62	0.95	1.38	0.490
Cer d33:1	2.01	1.85	1.91	0.490
DMPE 34:2 | DMPE 16:0_18:2	1.09	1.91	0.93	0.490
PC 40:4	0.94	0.65	1.46	0.490
PC P-40:6 or PC O-40:7 Isomer A	0.46	0.59	0.59	0.490
PE 38:4	1.34	2.21	1.39	0.490
PI 34:2 | PI 16:0_18:2	1.58	2.22	1.43	0.490
TG 46:0	0.87	0.54	0.53	0.490
TG 47:3;1O | TG 16:0_18:2_13:1;1O	0.94	0.70	0.49	0.492
PC P-36:3 or PC O-36:4	0.62	0.52	0.98	0.492
TG 55:3	1.59	0.98	0.72	0.492
FA 20:0 (arachidic acid)	0.70	1.32	1.44	0.494
TG 58:4	1.77	1.30	0.84	0.494
FAHFA 32:0 | FAHFA 16:0/16:0	0.47	0.62	0.79	0.496
LPC 20:2	0.81	0.60	1.28	0.496
PE 42:6	1.29	2.17	1.41	0.496
PG 44:12 | PG 22:6_22:6	0.48	0.89	0.79	0.496
TG 44:1	0.95	0.67	0.50	0.496
TG 59:3	1.80	1.42	0.89	0.496
PG 34:2 | PG 16:0_18:2	0.65	0.92	1.42	0.498
TG 58:5	2.03	1.49	1.11	0.503
LPC 17:1	0.52	0.70	0.50	0.504
PE P-38:5 or PE O-38:6	0.77	0.62	1.30	0.507
LPC 18:0_1	0.99	1.05	1.91	0.513
FA 20:1 (eicosenoic acid)	0.70	1.23	1.47	0.514
Cer d41:1	1.33	1.72	2.06	0.516
PC 38:1	0.54	0.50	0.60	0.516
PC 38:3	0.91	0.70	0.50	0.516
PE 34:2	0.50	0.85	0.62	0.516
PI 38:6 | PI 16:0_22:6	0.65	0.84	0.49	0.516
TG 48:3	1.10	0.69	0.56	0.516
PE 36:4 Isomer A	0.52	0.93	0.97	0.516

Cer 42:3;2O|Cer 18:2;2O/24:1	0.56	0.61	0.97	0.535
LPC 20:4_1	0.53	0.83	1.02	0.535
PC 35:4	1.75	1.95	1.79	0.535
PC P-38:4 or PC O-38:5 Isomer A	0.68	0.69	1.27	0.535
PC P-40:6 or PC O-40:7 Isomer B	1.99	1.62	1.83	0.535
PE 38:6 Isomer A	0.97	1.06	1.85	0.535
PE 40:6	0.91	1.68	0.86	0.535
PS 38:2	0.66	0.78	1.35	0.535
SL 33:0;2O|SL 17:0;O/16:0;O	0.50	0.58	0.57	0.535
SM d34:1	1.32	0.95	1.85	0.535
TG 56:4	1.78	1.34	0.91	0.535
LPC 18:0	1.06	1.07	1.91	0.537
PC 34:3	0.68	0.99	0.54	0.537
PC 35:3	1.35	2.06	1.24	0.537
CAR 18:1	1.56	1.51	2.07	0.550
FA 20:3 (eicosatrienoic acid)	0.54	0.76	1.03	0.550
TG 48:0|TG 16:0_16:0_16:0	0.84	0.55	0.56	0.554
PE 40:6_1	0.86	1.66	0.92	0.554
PC 34:2	1.09	1.77	0.91	0.560
PE 34:3	0.88	1.33	1.71	0.560
TG O-52:2|TG O-18:1_16:0_18:1	0.65	0.59	1.05	0.560
TG 62:3	1.25	0.76	0.65	0.570
TG 57:3|TG 21:0_18:1_18:2	1.50	1.09	0.74	0.572
TG 57:2|TG 23:0_16:1_18:1	1.45	1.15	0.73	0.573
TG 60:3	1.14	0.81	0.59	0.578
DG 34:3	0.64	0.75	0.51	0.588
SM d33:1	1.07	0.66	1.31	0.591
PE 38:1|PE 18:0_20:1	1.12	1.90	1.42	0.595
TG 52:0	1.88	1.13	1.10	0.595
PI 38:4|PI 18:0_20:4	0.51	0.79	0.67	0.597
PC 40:3	1.06	0.94	0.57	0.597
PE 38:4 Isomer B	1.14	1.87	1.09	0.598
PE 40:7|PE 20:3_20:4	0.70	0.78	0.51	0.600
TG 62:2	0.99	0.67	0.58	0.610
SM d41:1	0.96	1.03	1.72	0.628
CE 20:4	0.95	0.92	1.64	0.630
Cer d36:1	1.35	1.65	1.85	0.635
Cer 34:1;2O|Cer 18:1;2O/16:0	1.51	1.81	1.70	0.645
TG 62:4	1.18	0.95	0.63	0.658
PE P-34:1 or PE O-34:2	1.53	0.83	0.98	0.659
CE 22:2	0.84	0.89	1.50	0.661

FA 22:0 (behenic acid)	0.55	0.87	0.75	0.681
LPE 18:0	0.54	0.78	0.79	0.692
LPE 18:0_1	0.55	0.79	0.83	0.692
TG 60:5	1.74	1.20	1.05	0.692
TG 48:4 Isomer B	1.78	1.19	1.13	0.694
DG 38:5	1.51	1.69	1.69	0.696
LPC 15:0	0.64	0.87	1.14	0.707
DG 36:2	0.63	0.73	0.58	0.719
FA 20:5 (eicosapentaenoic acid)	1.10	1.74	1.20	0.719
PE 34:3 | PE 16:0_18:3	1.00	1.45	1.57	0.719
PE 36:4 | PE 18:2_18:2	0.80	1.41	1.19	0.719
PE 40:5	0.61	1.03	0.85	0.719
FA 22:6 (docosahexaenoic acid)	0.71	1.18	1.19	0.721
PE 37:5 | PE 17:1_20:4	1.00	1.01	1.62	0.721
CE 22:6	1.41	1.48	1.76	0.728
PS 38:4	1.35	1.70	1.59	0.728
SM d32:1	1.13	0.79	1.36	0.746
TG 46:3 Isomer A	0.66	0.59	0.70	0.749
PC 42:8	1.60	1.53	1.59	0.753
CE 22:5	0.69	0.70	1.05	0.760
PC 34:3 Isomer B	0.87	1.15	0.69	0.772
SM d34:2	0.61	0.65	0.76	0.774
PE 35:2 | PE 17:0_18:2	1.11	1.56	1.49	0.778
Cer d34:1	1.43	1.67	1.38	0.785
FA 20:4 (arachidonic acid)	0.76	1.10	1.27	0.786
PC P-32:0 or PC O-32:1	0.64	0.64	0.70	0.786
PS 40:6 | PS 18:0_22:6	1.24	1.58	1.51	0.796
PC 33:2_1	0.90	1.44	1.07	0.808
PC 36:4 Isomer A	1.23	1.58	1.08	0.818
CE 22:3	0.89	1.16	1.40	0.826
PC 37:5 Isomer A	1.37	0.91	1.28	0.828
DG 34:1	0.77	0.80	0.63	0.841
TG 60:8 | TG 18:1_18:1_24:6	1.53	1.15	1.39	0.841
DG 36:1	0.77	1.01	1.21	0.847
Cer d42:2 Isomer A	0.74	1.04	1.07	0.881
PC 39:5	1.11	1.05	1.47	0.881
PC 42:6 Isomer A	0.68	0.72	0.74	0.881
SM 34:2;3O	0.98	1.03	1.41	0.881
Cer 42:1;2O | Cer 18:1;2O/24:0	0.92	1.34	1.09	0.904
PC 40:5 Isomer A	1.32	0.94	1.11	0.928
CoQ9	0.92	1.15	1.28	0.928

PC 35:1	0.86	0.90	1.17	0.947
Cer d42:2 Isomer B	0.76	0.95	1.02	0.948
Cer d32:1	1.21	0.88	1.05	0.952
PE P-40:6 or PE O-40:7	1.18	0.93	1.20	0.956
PC P-40:5 or PC O-40:6	0.97	1.12	1.26	0.962
PS 38:6|PS 16:0_22:6	0.94	1.08	0.82	0.962
Cer d34:2	1.08	1.31	1.08	0.966
Cer d42:1	0.84	1.08	0.97	0.973
FA 15:0 (pentadecylic acid)	0.95	1.19	1.02	0.973
PC 36:4 Isomer C	1.01	1.24	1.13	0.973
PE 38:4 Isomer A	0.78	0.89	0.88	0.973
TG 46:3 Isomer B	1.11	1.01	1.23	0.973
CE 20:3	0.89	0.85	0.90	0.993
Cholesterol	0.87	0.90	0.91	0.994
PC 36:4 Isomer B	1.06	1.03	1.03	1.000

[a]Values are fold changes compared to the absolute control group that received normal-fat diets both prenatally and postnatally (NFCO-NF).

[b]P values are analyzed by ANOVA across different groups and adjusted for FDR using MetaboAnalyst 5.0.
NF, normal fat; CO, no choline supplement; HF, high fat; CS, choline supplement. FDR: false discovery rate
