# Supplementary material for: Prenatal Choline Supplement in a Maternal Obesity Model Modulates Offspring Hepatic Lipidomes
Source: Nutrients. 2023 Feb 15;15(4):965. doi: 10.3390/nu15040965 (PMC9963284; doi:10.3390/nu15040965)
Supplement: Supplementary file 1 [file nutrients-15-00965-s001.zip › nutrients-2176691-supplementary/Table S1_Lipidomics Paper.pdf]

Supplementary Table S1. Primers used for real-time PCR[1]

Gene Symbol	Forward Primer	Reverse Primer
Acer2	5′ ACAACGTGCTTGGCGTTTAT 3′	5′ CGCACATTGTCACACCTCTT 3′
Actb	5′ TAAGGCCAACCGTGAAAAGA 3′	5′ TGCCTGTGGTACGACCAGAG 3′
Agps	5′ ACGGTCAGAGGGGTTATTTGCT 3′	5′ AGATCTATAACCCTGTCCCAAG 3′
Ccl2	5′ ACCTGCTGCTACTCATTCAC 3′	5′ GAGCTTGGTGACAAAAACTACAG 3′
Cers2	5′ AGATGGACGTGTCTATGCCA 3′	5′ GGTGTTGCCACATAAAGCTC 3′
Col1a1	5′ CATGGCCAAGAAGACATCCC 3′	5′ CAGATCAAGCATACCTCGGG 3′
Cybb	5′ TTGCTGTGCACCATGATGAG 3′	5′ CGCCTATTGTGGTGTTAGGG 3′
Elovl1	5′ TGAGCTGATGGACACAGTGAT 3′	5′ TTTTATCCCCCACCACCAACT 3′
Fads2	5′ GCAGCCCCTTGAGTATGGCAAG 3′	5′ AGTACATAGGGATGAGCAGCGG 3′
Far1	5′ TTGAATGAAGAGATGTCTGGCC 3′	5′ GCGCCAGATAAGTATCACAAGG 3′
Gnpat	5′ AGATCCTCGGAGTTCCTAAGCC 3′	5′ AACGCAGTGATACAGGGTCTCC 3′
Gpx4	5′ TCTGTGTAAATGGGGACGATG 3′	5′ ACGCAGCCGTTCTTATCAAT 3′
Mfsd2a	5′ ACCCGGTTTGGCAAGAAGACAG 3′	5′ GGCCACCACGTAAGTGACGATT 3′
Sod	5′ CAGAAAACACGGTGGGCCAAAG 3′	5′ TGCAATGGTCTCCTGAGAGTGA 3′
Tnfa	5′ AGAAGTTCCCAAATGGCCTCCC 3′	5′ CGTTGGCCAGGAGGGCGTT 3′

[1]Abbreviations: *Acer2* (alkaline ceramidase 2), *Actb* (beta-actin), *Agps* (peroxisomal alkyldihydroxyacetonephosphate synthase), *Ccl2* [MCP-1/chemokine (C-C motif) ligand 2], *Cers2* (ceramide synthase 2), *Col1a1* (COL1A1 collagen type I alpha 1 chain), *Cybb* (cytochrome b-245 beta), *Elovl1* (elongation of very long chain fatty acids 1), *Fads2* (fatty acid desaturase 2), *Far1* (fatty acyl-CoA reductase 1), *Gnpat* (glyceronephosphate O-acyltransferase), *Gpx4* (glutathione peroxidase 4), *Mfsd2a* (mammalian family super domain 2 a), *Sod* (superoxide dismutase), *Tnfa* (tumor necrosis factor alpha).
